# Building protein structure-specific rotamer libraries

**DOI:** 10.1093/bioinformatics/btad429

**Published:** 2023-07-13

**Authors:** Algirdas Grybauskas, Saulius Gražulis

**Affiliations:** Sector of Crystallography and Cheminformatics, Institute of Biotechnology, Life Sciences Center, Vilnius University, 7 Saulėtekio Ave, Vilnius, LT- 10257, Lithuania; Sector of Crystallography and Cheminformatics, Institute of Biotechnology, Life Sciences Center, Vilnius University, 7 Saulėtekio Ave, Vilnius, LT- 10257, Lithuania

## Abstract

**Motivation:**

Identifying the probable positions of the protein side-chains is one of the protein modelling steps that can improve the prediction of protein–ligand and protein–protein interactions. Most of the strategies predicting the side-chain conformations use predetermined dihedral angle lists, also called rotamer libraries, that are usually generated from a subset of high-quality protein structures. Although these methods are fast to apply, they tend to average out geometries instead of taking into account the surrounding atoms and molecules and ignore structures not included in the selected subset. Such simplifications can result in inaccuracies when predicting possible side-chain atom positions.

**Results:**

We propose an approach that takes into account both of these circumstances by scanning through sterically accessible side-chain conformations and generating dihedral angle libraries specific to the target proteins. The method avoids the drawbacks of lacking conformations due to unusual or rare protein structures and successfully suggests potential rotamers with average RMSD closer to the experimentally determined side-chain atom positions than other widely used rotamer libraries.

**Availability and implementation:**

The technique is implemented in open-source software package *rotag* and available at GitHub: https://www.github.com/agrybauskas/rotag, under GNU Lesser General Public License.

## 1 Introduction

The essential step in predicting protein interactions with different proteins or ligands is analysis of the side-chain flexibility (DeLuca *et al.* 2015). There are multiple approaches how to tackle this problem, such as molecular dynamics (Das and Baker 2008), coarse-grain methods (Lombardi *et al.* 2016), or Monte Carlo sampling (Rohl *et al.* 2004). However, the most common approach in analysing and sampling side-chain positions is using rotational isomer (rotamer) libraries that are essentially lists of the most frequent side-chain dihedral angles ($\chi_{i}$) in protein structures (Dunbrack 2002).

The general technique of building rotamer libraries starts with selecting high-quality structures from the PDB (Berman *et al.* 2003). As the PDB has increased in size and quality over the years, more stringent criteria were used to include protein structures into the rotamer statistic datasets. The criteria mostly depended on the quantity of available crystal structures and the resolution of the solved protein structure and that would be the main parameter to filter by. The higher the resolution of the protein, the more localized atom positions are. The resolution threshold has become more stringent over the years due to improvements of experimental techniques and the increase of the number of entries in the PDB. Later, not only the average resolution was taken into account, but also the resolution of individual side-chains (Dunbrack 2002).

It was noticed that the diversity of the structures is essential for building rotamer libraries. Dunbrack and Karplus (1993) chose not to include identical structures and, later, Lovell *et al.* (2000) expanded on that idea not to include proteins that have sequence similarity >50%. Later, Scouras and Daggett (2011) (van der Kamp *et al.* 2010) used proteins with unique folds in order to get even more diverse datasets for building the rotamer library.

After choosing the initial set of protein crystal structures, side-chains were grouped by amino acid and, depending on the application of the library, by other criteria. Until sufficient quantity of protein structures were solved, all side-chain $\chi_{i}$ angles were analysed. With increasing number of protein crystal structures, additional criteria for clustering dihedral angles were used, such as protein secondary structure (McGregor *et al.* 1987), ranges of protein backbone ϕ and ψ angles (Dunbrack and Karplus 1993). Both backbone-independent (BBIND) and backbone-dependent (BBDEP) methods were the main ways to cluster side-chain angles, and both heavily depended on the quantity of initial protein structures.

Rotamer libraries can also vary in the ways how the most frequent dihedral angles are sampled from the statistical population. The common strategies are either using discrete or continuous generalization of the frequently occurring dihedral angles. Binning is one of the discrete methods to achieve the selection of the most frequent angles. Other common ways of selecting the most common angles are kernel density functions (Shapovalov and Dunbrack 2011) and Bayesian statistical analysis (Dunbrack and Cohen 1997). In addition, fully continuous model of sampling angles was also applied using dynamic Bayesian network (Harder *et al.* 2010).

Pre-calculated libraries from selected sets of protein structures are very convenient for side-chain prediction applications, because there is no need to regenerate them each time during the use. On the other hand, it was noticed that some flexible side-chains can end up having inaccurate models. For example, long chains, such as Arg, Glu, Gln, Lys and Met, when exposed on the surface of the protein, can be hard to fit to the electron density maps (Miao and Cao 2016). Even same proteins, but in different crystals, can show alternative side-chain dihedral angles (Miao and Cao 2016). This dihedral angle variety is lost when filtering out proteins with high-sequence similarity, because alternative side-chain positions might not be included (Miao and Cao 2016). Despite the fact that there are some rotamer libraries generated primarily from protein snapshots of molecular dynamics simulations (Towse *et al.* 2016), the above-mentioned methods still might lack the variety of the residues and their dihedral angles (Scouras and Daggett 2011, Towse *et al.* 2016).

It should be noted that deep-learning (DL) tools such as *AlphaFold2* (Jumper *et al.* 2021) for protein structure prediction or *DLPacker* (Misiura *et al.* 2022) for side-chain conformation prediction are becoming the methods of choice for modelling protein structures. However, rotamer libraries are still useful in certain cases. Rotamer libraries, unlike current implementations of DL methods, provide multiple conformation candidates for the residues of choice. The conformational variety that is present in rotamer libraries is necessary when modelling protein structures from X-ray crystallographic and cryo-EM density maps (Riley *et al.* 2021). Also, noncanonical amino acids that are rare in the PDB can be modelled with physics-based tools (Childers *et al.* 2018, Holden *et al.* 2022), whereas DL methods require large datasets of exemplary structures to produce the correct models. Lastly, results from DL-based methods are hard to interpret as learned parameters for the method are not necessarily constrained by the laws of physics. For example, it was noticed with *AlphaFold2* when trying to predict the impact of the point mutations to the protein stability, the energy values did not correlate with other widely used methods (Pak *et al.* 2023). Physics-based methods can yield insights into driving forces that shape side-chain conformations.

We suggest an approach to generating rotamer libraries that does not require collecting and filtering a set of protein structures. In our method, this is achieved by scanning through the conformational space of the specified side-chains and excluding dihedral angles that produce too high potential energies. The use of dead-end elimination (DEE) (Desmet *et al.* 1992) techniques and the fact that the longest side-chain has only 4 $\chi_{i}$ angles ensures that calculations do not reach combinatorial explosion. Although the use of predetermined angles is a faster approach, we argue that by dynamically scanning side-chains and calculating interactions between side-chains and protein backbone a greater conformational coverage will be achieved and more side-chains with rare conformations will be included.

## 2 Materials and methods

### 2.1 Extracting structural data from PDBx/mmCIF

To explore conformational space for every residue, the *rotag* software reads in initial structure of the residues from a PDBx/mmCIF (Bourne *et al.* 1997) format file (v5) as the main input. The main information for the program is located in atom_site data category in PDBx/mmCIF. Data items, such as group_PDB, id, type_symbol, label_atom_id, label_alt_id, label_comp_id, label_asym_id, label_seq_id, Cartn_x, Cartn_y, Cartn_z, pdbx_PDB_model_num, are mandatory, either because they identify atom types and coordinates or because they specify unique residues that the described atoms belong to. The *rotag* method uses backbone atom positions as local environment and side-chain atom positions as initial values for rotamer generation.

### 2.2 Determination of atom connectivities

Covalent bonds are determined in two ways: using an explicitly indicated list of chemical bonds in a force-field (FF) parameter file and using the grid-based cell-list neighbour finding algorithm, also called cubing procedure (Levinthal 1966). First, the program uses the list of bonds and, if it is unable to find the needed atom connections, uses the grid-based approach. The method uses cubing algorithm that has the cube edge length twice the largest covalent radius of the selected atoms. All covalent radii are provided in the FF parameter file and are based on the works of Pyykkö (Pyykkö and Atsumi 2009).

### 2.3 Scanning for conformational space of the side-chains

The conformational space search of the target side-chains is performed by first creating matrices that describe how the side-chain atom positions change depending on the values of dihedral angles, bond angles, and initial atom positions (Equation 1). The transformations of the atom positions are achieved by using homogeneous coordinates and 4×4 matrices containing rotational and translational components. These matrices first change frames of references so that rotating bond would be positioned along the *Z*-axis and then the rotation around the bond is applied. By storing pre-multiplied constant terms of the matrix equation (Equation 1), the new atom positions can be determined on demand saving CPU-time in the process. More thorough description of equation terms can be found in Supplementary Data (Supplementary Equations S1–S4). where $p^{0}$ initial atom coordinates in Cartesian frame; $p^{0}{}^{\prime }$ transformed atom coordinates in Cartesian frame; $T_{i-1}^{i}$ transformation matrix that changes one frame of reference to another; $R_{\chi_{i}}$ rotational matrix that changes the dihedral angle.


(1) $$p^{0}{}^{\prime }=T_{n}^{0}\cdot (\prod_{i=1}^{n}R_{\chi_{i}}\cdot T_{i-1}^{i})\cdot p^{0},$$

**Figure 1.**
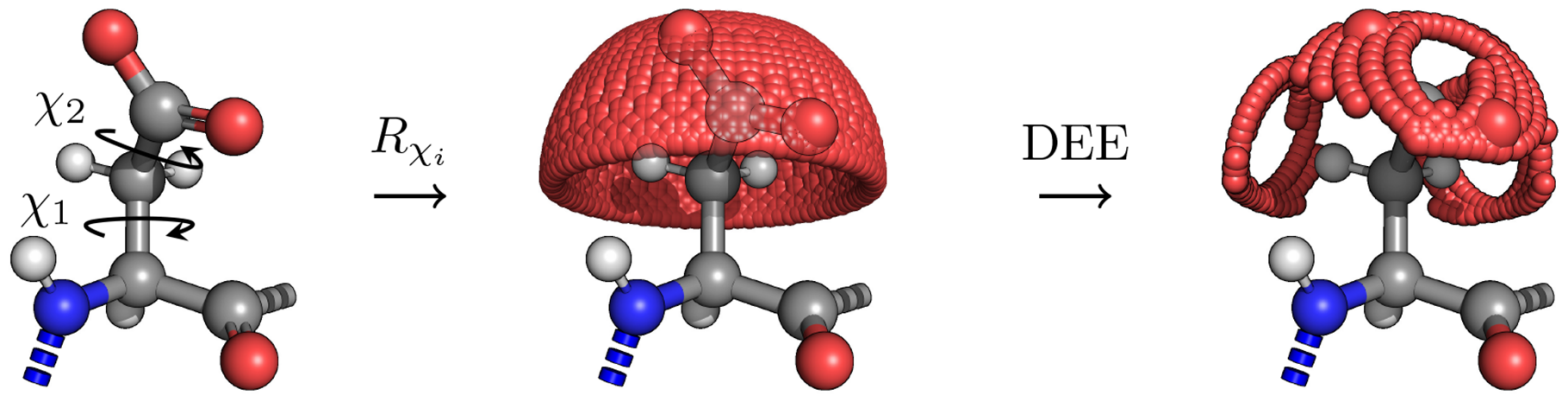
Simplified scheme for generating rotamer libraries.

**Figure 2.**
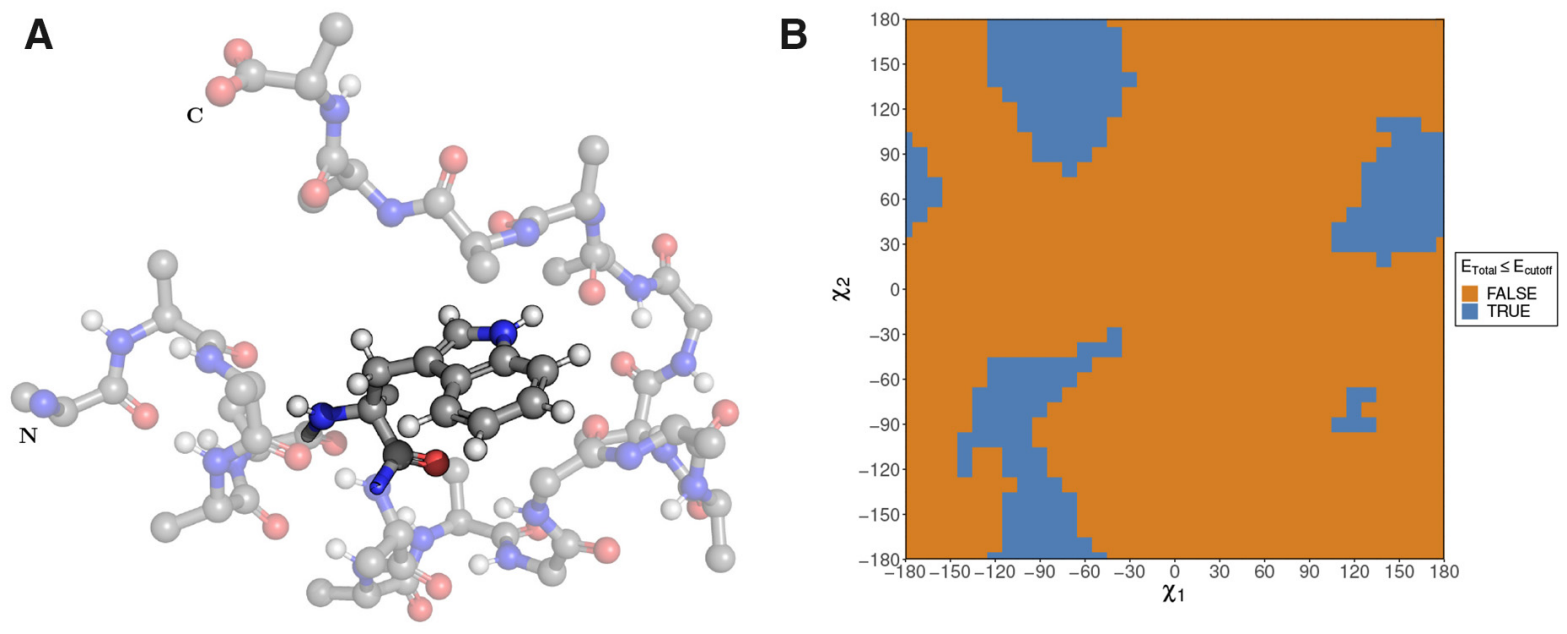
(A) Example of tryptophan interacting with spatially surrounded backbone atoms. (B) Energy-based selection condition for choosing rotamers. Blue colour depicts accepted dihedral angle pairs and orange—rejected.

Atom positions are being explored using dead-end elimination (Desmet *et al.* 1992) in order to minimize the amount of required computations. Side-chain atoms are added step-by-step and bond rotation (Equation 1, Fig. 1) is applied for each atom. Pairwise atom interaction energies are then calculated and summed to yield the total energy of the fragment analysed so far. It should be emphasized that calculations are performed not between two different residues, but between atoms of a single residue and mainchain atoms (with addition of CB) surrounding that residue (Fig. 2A).

When this energy exceeds the dead-end elimination threshold, the currently scanned dihedral angle is excluded from the further dihedral angle prediction steps. The dead-end threshold energy is selected by assuming that the rest of the uncalculated interactions will provide the best possible compensation of the energy growth. The final rotamers are selected from the energy landscape range that are below that energy threshold (Fig. 2B). In order to avoid $n^{2}$ complexity when calculating pairwise atom interactions, the same cubing procedure was adopted for the detection of neighbouring backbone and CB as when determining bond connections. The furthest distance that side-chain could reach from CA was assumed to be the length of the longest side-chain—ARG, with addition of appropriate Van der Waals radii and interaction boundary.

Almost all side-chains that have at least one $\chi_{i}$ dihedral angle were incorporated in the method. However, PRO was the exception. First of all, PRO is mostly found in one of the two conformations. Secondly, the way to get a proper conformational scan would be to include angle bending and length changing events and these features will only be adopted in the future.

### 2.4 Energy calculation

The total energy consists of the weighted sum of bonded and nonbonded energy potential functions. Nonbonded potential contains the weighted sum of Lennard-Jones, Coulomb and hydrogen bond potentials (Equation 2). *Amber18* force-field parameters were used for Lennard-Jones potential while Coulomb parameters were used from the modified version of the same *Amber18* (Maier *et al.* 2015). Smooth cutoff function (Supplementary Equation S9) was applied to avoid steep change of potential for Lennard-Jones, Coulomb and hydrogen bond potentials. Only torsional potential was used for the bonded potential term. However, it was slightly modified in order to adapt the potential to the dead-end elimination steps. Rather than having one continuous function, each term of dihedral angle turn is analysed in pairwise matter (Supplementary Equation S8). The weights and parameters for torsional potential were estimated by using particle swarm optimization (Kennedy and Eberhart 1995, Shi and Eberhart 1998) algorithm that tries to minimize RMSD of generated side-chain atom position models compared to selected reference PDB structures. where *E* energy value; *w* weight; *i*, *j* atom indexes; *d* dihedral angle index; *Q* distance cutoff function; LJ Lennard-Jones; C Coulomb; H hydrogen bond; T torsional.


(2) $$E_{\text{Total}}=\sum_{i}\sum_{j\neq i}Q_{ij}(w_{1}E_{ij}^{LJ}+w_{2}E_{ij}^{C}+w_{3}E_{ij}^{H})+\sum_{d}w_{4}E_{d}^{T},$$

### 2.5 Datasets

To test our rotamer generation procedure, eight datasets of observed residue structures were selected from the PDB (26 February 2022). First of all, high-quality structures were chosen, i.e. those with resolution ≤ 2.0 Å and $R_{\mathrm{free}}\leq$ 0.20 with sequence identity ≤70%, resulting in 9824 PDB structures. From these structures 1000 representative residues were selected randomly for each of 17 amino acids (20 amino acids from the standard genetic code except GLY, ALA, PRO) for each dataset. Multiple datasets were constructed so that we could check whether each dataset is representative of the residue population in the PDB.

### 2.6 Best-case RMSD and dihedral angles

In order to test *rotag* performance against other widely used rotamer libraries, comparable libraries were generated using *rotag*. These *rotag* libraries were compared against Dunbrack BBDEP (Shapovalov and Dunbrack 2011), Dynameomics BBDEP and BBIND (Towse *et al.* 2016), and Ultimate BBIND libraries (Hintze *et al.* 2016). Dunbrack and Ultimate rotamer libraries are based on high-quality datasets of full PDB structures with different approaches on the dihedral angle sampling while the dataset of Dynameomics method consists of dihedral angle occurrences in molecular dynamics simulations performed on unique protein folds. The comparison consisted of calculating best-case RMSD (bcRMSD) and best-case dihedral angles (bcDA) (see Supplementary Section S1.3) of each library against experimental data from the PDB. These two parameters were chosen for the study, because we want to evaluate the libraries from the protein modeller’s viewpoint. The idea is to find the library that would include rotamers with atom positions as close to those of the experimental dataset as possible.

The statistics for comparing bcRMSD were chosen such that the nonnormal distributions would be analysed. For this purpose, Wilcoxon signed-rank test was chosen [R package: *stats* (https://www.R-project.org/)]. The test is unhelpful by itself without the knowledge of the magnitude of two measurement differences with respect to their standard deviations. Therefore, the effect size or Cohen’s D parameter was also calculated [R package: *effsize* (https://CRAN.R-project.org/package=effsize)].

The structures with bcRMSD higher than 0.1 Å were selected for further analysis. Such bcRMSD cutoff value was chosen from experience as significant for protein modelling applications. It is also supported by values obtained from Luzzati distribution (Luzzati 1952) that describes statistical errors of structure factors and implies that all of the atoms are subject to coordinate errors. The average estimated coordinate error was calculated from the Luzzati plot for high-quality structures (see Section 2.5) and yielded value 0.16 ± 0.06 Å, which is in good correlation with the chosen bcRMSD cutoff. From structures with bcRMSD ≥0.1 Å, predicted dihedral angles that differ more than 10° from the corresponding experimental ones were analysed. The difference of 10° was selected, because for a dihedral angle in an *–$sp^{3}$–$sp^{3}$–* (‘*’ denotes any atom in any hybridization) side-chain fragment such change in the angle induces roughly 0.1 Å shift in the most distant atom.

### 2.7 Side-chain symmetry

**Figure 3.**
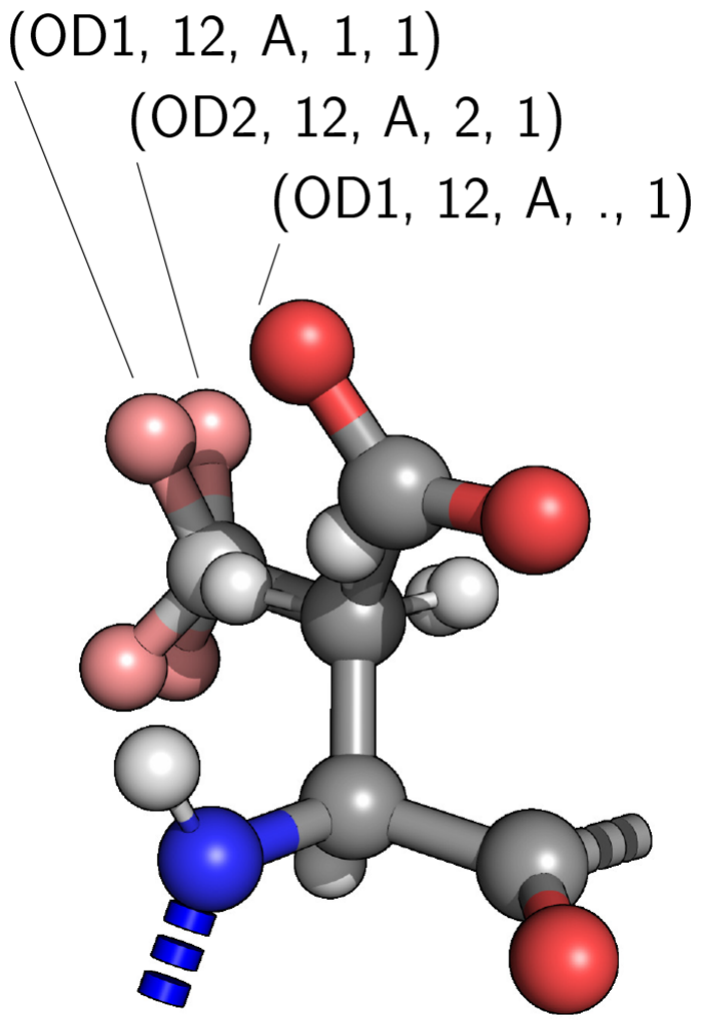
The example of ASP $\chi_{2}$ dihedral angle symmetry.

The calculation of bcRMSD requires that the atom positions and the atoms themselves from the selected rotamer library could be comparable to those from experimental data. Sometimes, side-chain atoms in crystallographic data are indistinguishable if the resolution of the structure is not high enough or if chemical group is symmetrical. ASP is an example of such amino acid where $\chi_{2}$ is indistinguishable from $\chi_{2}$ + 180° in the deprotonated form of the residue (Fig. 3). By reducing the range of required dihedral angles, the amount of calculations required to scan all side-chain positions is lowered. Only range from –90° to 90° is required for $\chi_{2}$ angle in order to get full range of angle coverage. The same situation applies to $\chi_{2}$ angles of PHE, TYR and $\chi_{3}$ angles of GLU. In electron density maps of protein X-ray structures, amino group and carbonyl oxygen are often hard to distinguish. Therefore, for the benchmarking purposes only, ASN $\chi_{2}$ and GLN $\chi_{3}$ dihedral angles are compared in $\left[-90^{{}^{\circ}},90^{{}^{\circ}}\right]$ range, neglecting the differences between the carbonyl oxygen and amide nitrogen. Nevertheless, when using *rotag* to search for side-chain positions, ASN $\chi_{2}$ and GLN $\chi_{3}$ dihedral angles are fully scanned—from –180° to 180°.

### 2.8 Rotamer count

Ideally, the best rotamer generation method should produce rotamers with atom positions as close to experimental positions as possible with the least overall choice count. By scanning all the possible side-chain conformations using user-defined dihedral angle change, the minimal bcRMSD value could be produced. However, the amount of dihedral angles to choose from would be computationally excessive in side-chain structure prediction applications if the dihedral angle range are too finely divided. Side-chains such as ASP, ASN, GLU, GLN, PHE, TRP, HIS, and TYR have dihedral angle distributions with high standard deviations near local maxima and for this reason lack the discrete identifiable rotamer angles (Shapovalov and Dunbrack 2011). Moreover, side-chain dihedral angle distributions differ for residues that are buried inside protein core or exposed on the protein surface. Thus, for each side-chain case, rotamer count is also included into analysis.

### 2.9 Rotamer library output

The output for rotamer angles, energy potential values, and atom coordinates of both *rotag* and pre-calculated rotamer libraries are stored in PDBx/mmCIF format (Supplementary Figs S6–S8).

### 2.10 Data visualization

R packages were used to visualize output data [*ggplot2* (Wickham 2011), *ggvenn* (Yan 2021), *ggrepel* (Slowikowski 2018), *stringi* (Gagolewski 2022), *RColorBrewer* (Neuwirth and Neuwirth 2014), *readr* (Wickham *et al.* 2015)] and further compare the statistics [*mixtools* (Benaglia *et al.* 2010), *Metrics*, *pwr*]. *PyMOL* (DeLano *et al.* 2002) and *Jmol* (Hanson 2010) was used to inspect actual side-chain positions.

## 3 Results

### 3.1 bcRMSD and bcDA comparisons

The analysis of bcRMSD and bcDA revealed interesting differences between rotamer libraries for some side-chains. Because the rotamer libraries generated with *rotag* are backbone-dependent, most of the analysis in this article will be related to BBDEP libraries. Nonetheless, useful statistics and observations of BBIND rotamer libraries will be mentioned.

**Figure 4.**
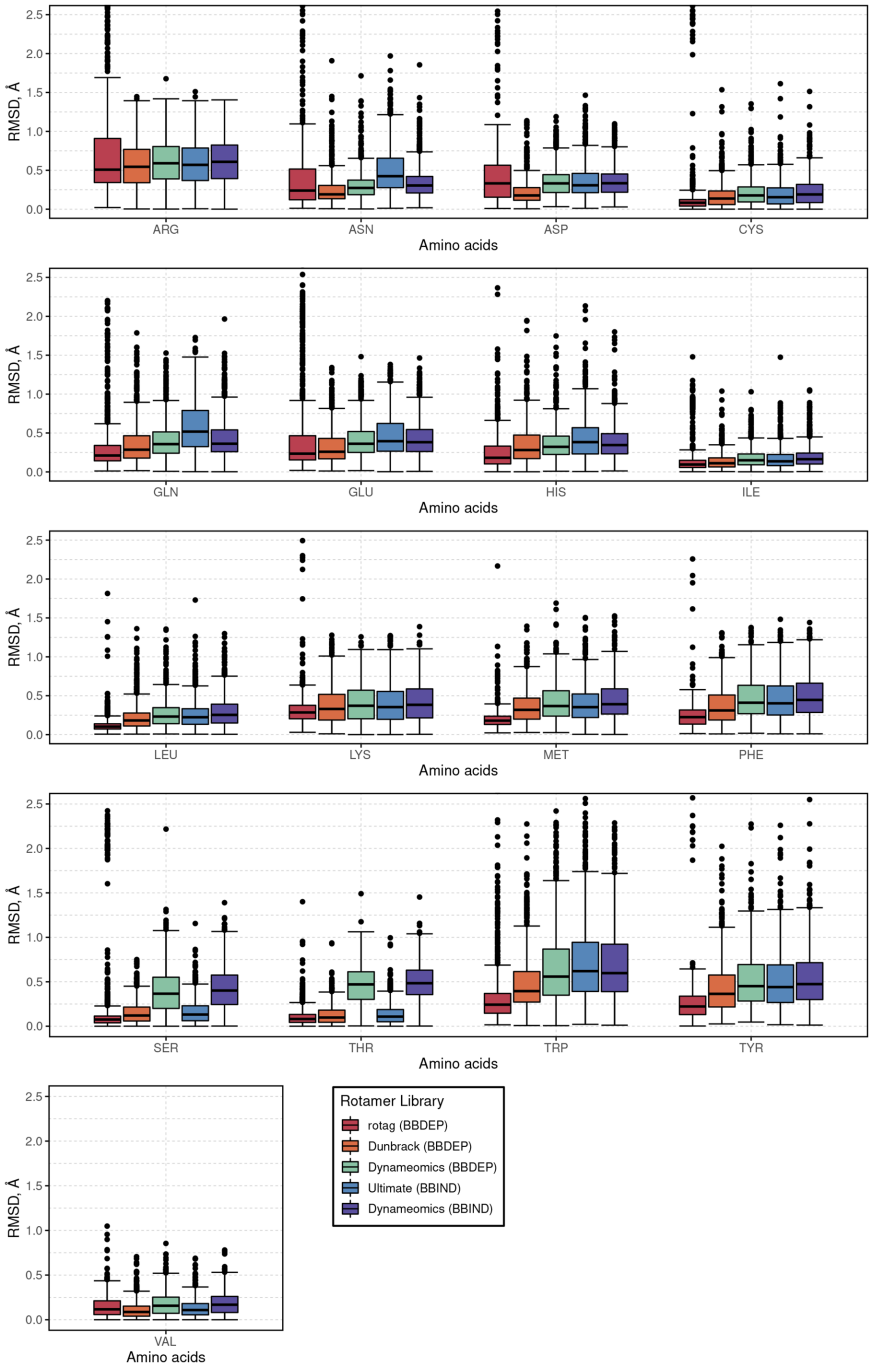
bcRMSD for all rotamer libraries.

In the present analysis, absolute difference of bcRMSD medians with values ≥0.10 Å and Cohen’s D absolute values ≥0.50 were chosen as significant. Two amino acids, LEU and MET, turned out to have paired median bcRMSD absolute differences (with 95% CI) of 0.092±0.013, 0.166±0.017 Å and paired absolute Cohen’s D of 0.575, 0.912, respectively, that are significant according to the above-mentioned criteria (Fig. 4 and Supplementary Table S2). The smaller bcRMSD values of *rotag* suggest a slight advantage in average accuracy for the *rotag* generated libraries.

Pairwise comparisons between *rotag* and Dunbrack library for the identified amino acids, LEU (Supplementary Fig. S23) and MET (Supplementary Fig. S28), show that in Dunbrack BBDEP $\chi_{i}$ angle spread around the identified distribution modes has smaller standard deviations than the spread observed in experimental structures. For example, LEU $\chi_{1}$ angle density has two distinct peaks for Dunbrack rotamer library case (Supplementary Fig. S23)—at 179.60° and –64.48°. The positions of those peaks are close to the distributions of produced by experimental data, however, the standard deviation is noticeably smaller—σ=2.66 Å and σ=3.86 Å for Dunbrack and σ=7.46 Å and σ=8.85 Å for experimental angle distributions. The smaller standard deviations around the distribution mode peaks could explain the lack of dihedral angles when picking rotamers from Dunbrack BBDEP library.

The bcRMSD and bcDA analysis for Dynameomics BBDEP and BBIND revealed insufficient rotamer choices. Because Dynameomics BBIND is a subset of Dynameomics BBDEP, BBDEP was analysed further. For example, $\chi_{2}$ gaps of $\left[-60^{{}^{\circ}},-30^{{}^{\circ}}\right]$ and $\left[30^{{}^{\circ}},60^{{}^{\circ}}\right]$ were observed in PHE (Supplementary Fig. S34) and TYR (Supplementary Fig. S49) bcDA distributions in Dynameomics BBDEP whereas *rotag* has dihedral angles in these ranges (Supplementary Figs S32 and S47). It should be noted that the same absence of angles in Dynameomics BBDEP library was noted in the original article (van der Kamp *et al.* 2010) and ascribed to nonrotameric amino acids. The absence of these crucial angles can introduce errors in the overall model during the side-chain modelling process. Likewise, the MET $\chi_{3}$ dihedral angle distribution in Dynameomics rotamer libraries has lower standard deviations than in experimental data (Supplementary Fig. S29). Moreover, two side-chains with single dihedral angle SER (Supplementary Fig. S39) and THR (Supplementary Fig. S44) had shifted bcDA in Dynameomics BBDEP subset. For SER and THR in this subset, *gauche-* conformation is shifted by +15°, *gauche+* is shifted by –20° and *trans—*by +10° (except for THR) with regards to experimental data. The last amino acid with discrepancy on bcDA is LEU. Interestingly, angle distribution modes of Dynameomics BBDEP are aligned with experimental values, but the difference of dihedral angle variances is noticeable (Supplementary Fig. S24) and $\chi_{2}$ dihedral angles from –60° to 45° lacked rotamers to choose from.

With regards to Ultimate BBIND rotamer library, most of the same problematic amino acids: GLN, MET, LEU and PHE have even greater bcRMSD (Supplementary Table S2). However, this is expected since the Ultimate BBIND rotamer library contains fewer rotamer choices for these residues.

Even though the rotamer libraries generated with *rotag* show overall better bcRMSD results, there are some angle deviations from experimental data that should be mentioned. $\chi_{2}$ angles are shifted by 30° for ASP (Supplementary Fig. S12) and 15° for ASN (Supplementary Fig. S17). Such shifts might be due to inaccuracies in force field parameters.

### 3.2 Rotamer count

One of the criteria for rotamer libraries that are suited for both solving crystal structures and predicting protein models is the amount of available rotamers. As mentioned in methods, one could argue that all angles at 10° change could be included to the library. However, during the protein structure prediction process this approach could lead to the combinatorial explosion. So, a good rotamer library should have balanced number of rotamer choice. The average quantity of rotamers (Table 1) suggests that *rotag* has greater number of choices for most residues, exceptions being ARG, PHE, TRP, THR, TYR, and VAL. Rotamer counts for LYS, MET are in hundreds—246 and 258 respectively. Although the number of suggested rotamers for these side-chains is high, it should be noted that proteins usually do not consist of only these types of amino acids, therefore large amount of rotamers would rarely be calculated for each residue. If large number of rotamers is a problem for a given application, number can be reduced by filtering out rotamers with high potential energy values in *rotag* library. However, the current implementation of *rotag* is designed to include slightly more rotamers, so that applications can choose at which energy level should excessive rotamers be excluded.

**Table 1.** Average number of rotamer choices for each side-chain per rotamer library.

Rotamer Library	ARG	ASN	ASP	CYS	GLU	GLN	HIS	ILE	LEU
Dunbrack (BBDEP)	75	36	18	3	54	108	36	9	9
Dynameomics (BBDEP)	76	17	9	3	27	51	17	9	9
Dynameomics (BBIND)	81	18	9	3	27	54	18	9	9
rotag	47	11	15	4	68	75	12	17	13
Ultimate (BBIND)	60	5	4	3	9	13	8	7	8

	**LYS**	**MET**	**PHE**	**SER**	**TRP**	**THR**	**TYR**	**VAL**	

Dunbrack (BBDEP)	73	27	18	3	36	3	18	3	
Dynameomics (BBDEP)	73	26	6	3	9	3	6	3	
Dynameomics (BBIND)	80	27	6	3	9	3	6	3	
rotag	246	258	1	6	2	2	1	2	
Ultimate (BBIND)	46	23	4	3	7	3	4	3	

### 3.3 bcRMSD distribution per rotamer library

**Figure 5.**
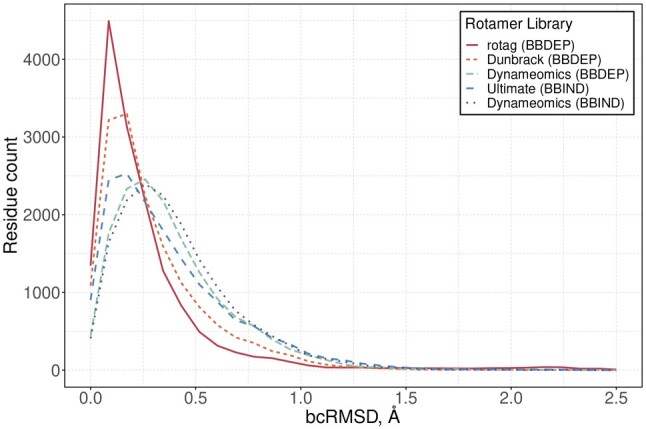
bcRMSD distribution.

The calculated overall distribution of bcRMSD displayed the average performance of rotamer library. The amount of side-chains that had certain bcRMSD values showed that using *rotag* could potentially produce the largest amount of side-chains with bcRMSD <0.1 Å (Fig. 5). Dunbrack BBDEP library would have the second largest amount of side-chains with low bcRMSD values. Ultimate BBIND, Dynameomics BBDEP and Dynameomics BBIND would tend to have more residues in higher bcRMSD range. The interesting observation is that Dynameomics BBDEP and BBIND would produce almost identical curves. However, it would be due to the fact that Dynameomics BBIND was created from Dynameomics BBDEP subset (Towse *et al.* 2016).

## 4 Discussion

The difference in dihedral angle by 10° for side-chain could seem as a minuscule one. However, changes in subsequent $\chi_{i}$ angles can have cumulative effect on the position of terminal side-chain atom. It is, of course, possible that the changes in $\chi_{i}$ angles partially compensate the changes in positions in terminal atoms. Nonetheless, 10° or 0.1 Å in the context of atom interactions should not be ignored, because interactions such as hydrogen bonding (Supplementary Equation S7) are very sensitive to the angle and distance change. Intermolecular forces, such as van der Waals (Supplementary Equation S5) or ionic interactions (Supplementary Equation S6) that are nondirectional, can be influenced greatly by the change of the distance between interacting atoms if there is an alternative side-chain position with lower potential energy. Finally, the dihedral angle change of 10° itself influences the distance of interacting atoms.

**Figure 6.**
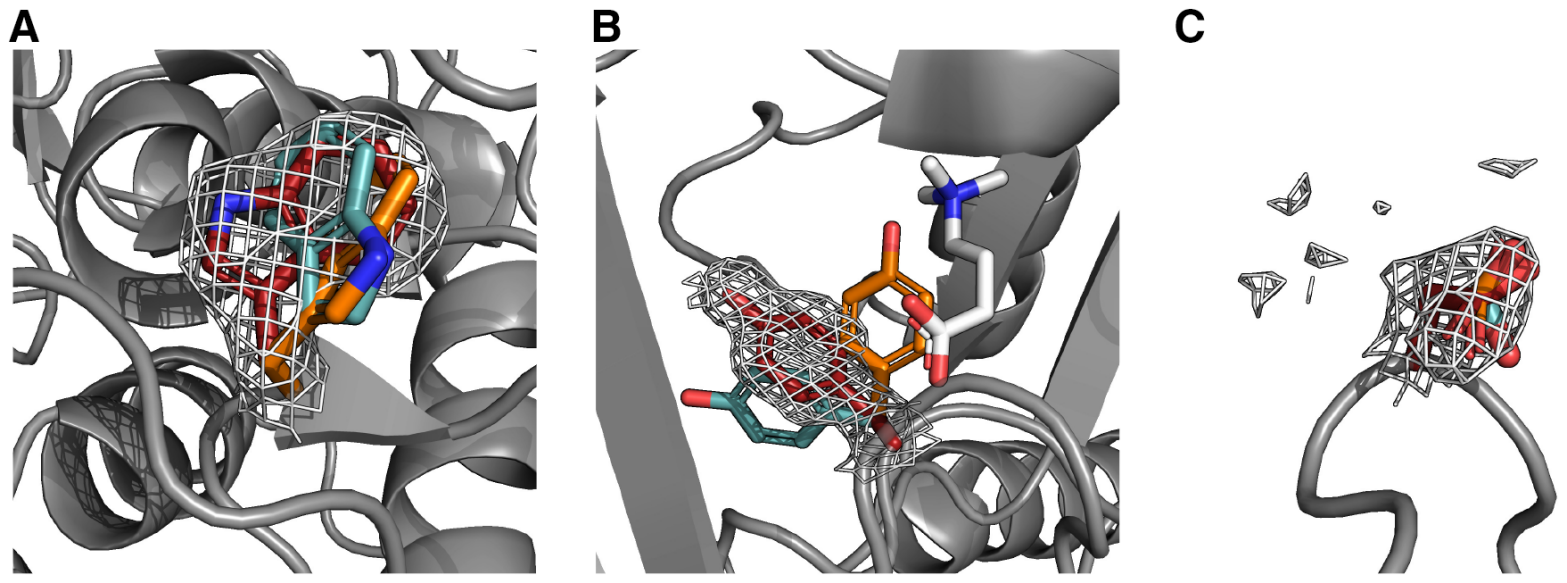
BBDEP rotamers placed in the electron density maps: (A) W391 in 2ww2_C. (B) Y205 in 4c5w_A in. (C) N47 in 4o6s_A. Red side-chains represent rotamers from *rotag* library, orange—Dunbrack, cyan—Dynameomics and white or gray—atoms and proteins from PDB structures.

Analysis of the rotamer libraries added a broader view of how atom positions of certain side-chains might vary. Although the currently widely used rotamer libraries, such as Dunbrack BBDEP, Dynameomics BBDEP and BBIND, and Ultimate BBIND represent only the most frequent averaged angles, it should be stressed out that outliers should not be ignored. The outlier angles are significant especially when specific regions like active or binding sites or protein–protein interaction interfaces are present (Hintze *et al.* 2016). The outliers might occur due to the existence of ligands (Fig. 6B), hindrances of other neighbouring atoms (Fig. 6A) or possible alternative positions of the side-chains (Fig. 6C). Ignoring such angles might lead to overlooked important interactions between side-chains and/or ligands. Widely used rotamer libraries like Dunbrack or Ultimate are created by taking crystal structures and offering representative average dihedral angles from the conformation space of the side-chains. Working with averaged values might exclude side-chain conformations that are outliers due to atypical interactions. Even though *rotag* generated libraries or simulated ones with Dynameomics methodology are unable to capture suitable rotamers for some outliers (Supplementary Fig. S11), other outlier angles are better represented using *rotag*. Therefore, *rotag* could be used as an alternative or additional rotamer library to the existing ones.

Rare dihedral angles in PDB is not the only problem caused due to the lack of occurrences. Side-chains, such as selenocysteine or pyrrolysine are very rare (61 and 2 occurrences respectively) in PDB as of 26 February 2022, so the sample size of these side-chains are insufficient for building rotamer libraries. Methods that were used in building Dynameomics library could be applied, but the actual libraries were not built. Using *rotag* software would be an option to accomplish this task, because it only requires single protein structure with available explored side-chain coordinates during the scan of conformational space.

## 5 Conclusion

The main idea of creating rotamer library that could be generated for the specific protein structure was to avoid averaging problems from pre-calculated side-chain dihedral angle datasets. Outliers are important especially when they are in conformationally restricted positions in active sites or protein–protein interfaces. Since *rotag* calculations are specific for the target protein structure, they are dynamic, scanning angles can be changed, adjusted, and recalculated. Also, *rotag* removes impossible conformations right away. During the selection of rotamers, *rotag* helps to address the nonrotameric conformation problem (van der Kamp *et al.* 2010), because it is possible to choose from the whole distribution of dihedral angles. The *rotag* comparisons with other widely used rotamer libraries showed its capability to have more accurate results when dealing with outliers. Although for some side-chains, such as LYS and MET *rotag* might offer excessive rotamer choices or prolonged dihedral angle calculations for ARG, GLN, ILE, LEU, LYS, and MET (Table 2), the excess rotamer choices can be filtered out by potential energy value cutoff and the calculations can be sped up by decreasing dihedral angle scanning step count. The *rotag* could be used along the existing rotamer libraries where critical amino acids are being thoroughly studied in order not to miss important rare rotamers.

**Table 2.** Average calculation time for single amino acid (sample size per amino acid N=30).^a^

Residue	Time (s)	Residue	Time (s)	Residue	Time (s)
ARG	31.25	HIS	12.30	SER	7.70
ASN	7.40	ILE	29.17	THR	8.40
ASP	5.24	LEU	31.92	TRP	13.96
CYS	8.47	LYS	25.59	TYR	7.27
GLN	29.00	MET	33.01	VAL	11.43
GLU	19.76	PHE	8.28		

a Calculations were performed on the computer with AMD Ryzen 7 1800X 8-core processor (16 threads) and 64 GB DDR4 RAM.

One of the greater advantages of the method used in *rotag* is its ability to deal with nonstandard amino acids. There are only a handful of structures with selenocysteine or pyrrolysine in the PDB which precludes collection of extensive statistics. This is, however, not a problem for *rotag* since its method does not require pre-calculated averaged dihedral angle datasets.

In the future, bond angle bending and bond length change will be included as additional degrees of freedom in order to get even more diverse possibilities of side-chain conformations. This type of rotamer library generation method is a first step for developing side-chain positions solver independent of pre-calculated rotamer libraries that could be used in the protein–protein or protein–ligand docking.

## Supplementary Material

btad429_Supplementary_Data
