# Supplementary material for: Building protein structure-specific rotamer libraries
Source: Bioinformatics. 2023 Jul 13;39(7):btad429. doi: 10.1093/bioinformatics/btad429 (PMC10359632; doi:10.1093/bioinformatics/btad429)
Supplement: btad429_Supplementary_Data [file btad429_supplementary_data.zip › btad429_Supplementary_Data.pdf]

Supplementary Data

Contents

1 Equation terms of conformational model **2**

2 Equation terms of energy potential **2**

3 bcRMSD and bcDA **3**

4 CIF input/output **4**

4.1 _atom_site 4
4.2 _[local]_rotamer_angle 6
4.3 _[local]_rotamer_energy 7

5 Basic usage cases **8**

6 Best-case RMSD **9**

6.1 Raw data set results 9

7 Best-case RMSD statistics **10**

7.1 Data set 13097 10
7.2 Data set 16226 12
7.3 Data set 15222 14
7.4 Data set 16089 16
7.5 Data set 16931 18
7.6 Data set 19153 20
7.7 Data set 25759 22
7.8 Data set 29821 24

8 bcRMSD outliers **26**

8.1 Data set 13097 26
8.2 Raw data set results 26

9 Best-case dihedral angles **27**

9.1 Data set 13097 27
9.1.1 ASP 27
9.1.2 ASN 29
9.1.3 LEU 31
9.1.4 MET 34
9.1.5 PHE 40
9.1.6 SER 42
9.1.7 THR 44
9.1.8 TYR 46
9.2 Raw data set results 48

1 Equation terms of conformational model

The atom positions (x, y, z) are stored and operated in Cartesian coordinate system where the units of Å are chosen as in PDBx/mmCIF '_atom_site' data category. The initial atom positions are represented in vector form $\boldsymbol{p}^0$ (Eq. S1) which rotational and translational transformations are applied to during the bond rotations in the side-chains and converted to the final vector $\boldsymbol{p}^{0\prime}$ (Eq. S2).

$$\boldsymbol{p}^0 = \begin{bmatrix} x \\ y \\ z \end{bmatrix} \quad \text{(S1)} \qquad \boldsymbol{p}^{0\prime} = \begin{bmatrix} x' \\ y' \\ z' \end{bmatrix} \quad \text{(S2)}$$

The first step of the conversion from $\boldsymbol{p}^0$ to $\boldsymbol{p}^{0\prime}$ is to change the frame of reference of the coordinate system such that rotation would occur around bond by χ_i degrees on local reference frame Z axis. α, β and γ correspond to Euler angles that are required to convert from $i-1$ frame of reference to i. This frame of reference transformation is performed with $\boldsymbol{T}^i_{i-1}$ 4×4 matrix (Eq. S3).

$$\boldsymbol{T}^i_{i-1} = \begin{bmatrix} cos(-\gamma) & -sin(-\gamma) & 0 & 0 \\ sin(-\gamma) & cos(-\gamma) & 0 & 0 \\ 0 & 0 & 1 & 0 \\ 0 & 0 & 0 & 1 \end{bmatrix} \begin{bmatrix} 1 & 0 & 0 & 0 \\ 0 & cos(-\beta) & -sin(-\beta) & 0 \\ 0 & sin(-\beta) & cos(-\beta) & 0 \\ 0 & 0 & 0 & 1 \end{bmatrix} \begin{bmatrix} cos(-\alpha) & -sin(-\alpha) & 0 & 0 \\ sin(-\alpha) & cos(-\alpha) & 0 & 0 \\ 0 & 0 & 1 & 0 \\ 0 & 0 & 0 & 1 \end{bmatrix} \begin{bmatrix} 1 & 0 & 0 & -x_i \\ 0 & 1 & 0 & -y_i \\ 0 & 0 & 1 & -z_i \\ 0 & 0 & 0 & 1 \end{bmatrix} \quad \text{(S3)}$$

After the change of the frame of reference and Z axis is on the bond that has to be rotated, the rotation is applied with $\boldsymbol{R}_{\chi_i}$ (Eq. S4):

$$\boldsymbol{R}_{\chi_i} = \begin{bmatrix} cos(\chi_i) & -sin(\chi_i) & 0 & 0 \\ sin(\chi_i) & cos(\chi_i) & 0 & 0 \\ 0 & 0 & 1 & 0 \\ 0 & 0 & 0 & 1 \end{bmatrix} \quad \text{(S4)}$$

After all the rotations, the frame of reference has to be brought back to the origin by $\boldsymbol{T}^0_n$.

2 Equation terms of energy potential

The composite energy potential used in *rotag* is a linear combination of Lennard-Jones (Eq. S5), Coulomb (Eq. S6), hydrogen bonding (Eq. S7) and torsion potentials (Eq. S8).

The standard Lennard-Jones potential [Jones, 1924] was used for electrically neutral atom interactions. σ^{LJ}_{ij} and ϵ^{LJ}_{ij} parameters of *Amber18* [Maier et al., 2015] were chosen. r_{ij} represents the distance between atoms.

$$E_{ij}^{LJ} = 4 \cdot \epsilon_{ij}^{LJ} \cdot \left[\left(\frac{\sigma_{ij}^{LJ}}{r_{ij}} \right)^{12} - \left(\frac{\sigma_{ij}^{LJ}}{r_{ij}} \right)^{6} \right] \tag{S5}$$

The partial charge parameters from *Amber18* for electrostatic potential was also used.

$$E_{ij}^{C} = \frac{q_i \cdot q_j}{r_{ij}^2} \tag{S6}$$

In order to have more detailed description of hydrogen bonding, AutoDock4 [Morris et al., 2009] term and force-field parameters were used for this purpose. Because hydrogen bond depends both on angle and distance between atoms, additional θ_{ijk} angle exists. It covers the angle between two atoms and hydrogen in between.

$$E_{ijk}^{H} = \epsilon_{ij}^{H} \cdot \left[5 \cdot \left(\frac{\sigma_{ij}^{H}}{r_{ij}} \right)^{12} - 6 \cdot \left(\frac{\sigma_{ij}^{H}}{r_{ij}} \right)^{10} \right] \cdot cos(\theta_{ijk}) \tag{S7}$$

In order to simplify the calculations of torsion angles and isolate the torsion potential between four atoms, the piecewise curves were applied. Parameters from *Amber18* again were used to incorporate the amplitudes ϵ_{ijkl}^{T} from the force field (Eq. S8). ϕ represents the parameter that manipulates the width of potential peak between torsion atoms i and l and γ represents the angle shift.

$$E_{ijkl}^{T} = \begin{cases} 0, & \text{if } \frac{\pi+\gamma}{\phi} < \omega_{ijkl} < \frac{-\pi+\gamma}{\phi} \\ \frac{\epsilon_{ijkl}^{T}}{2} \cdot (1 + cos(\phi \cdot \omega_{ijkl} - \gamma)) \end{cases} \tag{S8}$$

In order not to calculate Lennard-Jones potential for all atoms, cutoff distance had to be chosen. However, if the cutoff is just a simple constant, the steep drop of the potential function can produce strange results so, a cutoff function was used (Eq. S9). The parameter σ_{ij}^{LJ} is the same as in Lennard-Jones potential and c parameters define when the smooth transition to zero value starts with respect to VdW radii of interacting atoms.

$$Q_{ij} = cos \left(\frac{\pi \cdot (r_{ij} - c_{start} \cdot \sigma_{ij}^{LJ})}{2\sigma_{ij}^{LJ} \cdot (c_{end} - c_{start})} \right) \tag{S9}$$

3 bcRMSD and bcDA

A rotamer can be described by a list of dihedral angles DA_{n_s}, where n_s is the number of rotating bonds for a side-chain (Eq. S10). Therefore, a rotamer library is just a collection of those rotamer dihedral angles DA_{n_r} (Eq. S11) for the given side-chain, where n_r is the number of rotamers in the rotamer library.

$$DA_{n_s} = (\chi_1, ..., \chi_{n_s}) \tag{S10}$$

$$DA_{n_s,n_r} = ((\chi_{1,1}, ..., \chi_{n_s,1}), ..., (\chi_{1,n_r}, ..., \chi_{n_s,n_r})) \tag{S11}$$

$$\boldsymbol{P}_{n_a} = (\boldsymbol{p}_1^0, ..., \boldsymbol{p}_{n_a}^0) \tag{S12}$$

In order to calculate bcRMSD parameter for the rotamer library of the selected side-chain, each list of rotamer dihedral angles in the rotamer library have to be compared against the dihedral angles calculated from the experimental structure (see Algorithm 1). First, list of dihedral angles in the rotamer library is used to convert side-chain atom coordinates of the X-ray structure $\boldsymbol{p}_k^0$ to the atom coordinates of the rotamer $\boldsymbol{p}_k^{0'}$ by applying Eq. 1 iteratively, where n_a is the number of atoms in the side-chain. In the Algorithm 1 this function is denoted as P. After that atom coordinates for each rotamer are compared against the atom positions in the structural data using RMSD calculations. The smallest RMSD value is called bcRMSD (best-case RMSD) and the dihedral angles producing that value – bcDA (best-case dihedral angles).

Algorithm 1 bcRMSD calculation algorithm

procedure BCRMSD($\boldsymbol{P}_{n_a}$, DA_{n_s,n_r})
    $bcRMSD^2 \leftarrow \infty$
    for $j \leftarrow 1, n_r$ **do** ▷ Iterates through all rotamers.
        $RMSD^2 \leftarrow 0$ ▷ Accumulates squared differences over n_a.
        ▷ Will contain squared RMSD at the end of the following two loops.
        for $k \leftarrow 1, n_a$ **do** ▷ Iterates through all atoms.
            for $i \leftarrow 1, n_s$ **do** ▷ Iterates through all rotating bonds.
                $\boldsymbol{p}_k^{0'} \leftarrow P(\boldsymbol{p}_k^0, DA_{i,j})$ ▷ Applies rotation around bond.
                $RMSD^2 \leftarrow RMSD^2 + \frac{(\boldsymbol{p}_k^{0'} - \boldsymbol{p}_k^0)^2}{n_a}$
            end for
        end for
        if $bcRMSD^2 \geq RMSD^2$ **then**
            $bcRMSD^2 \leftarrow RMSD^2$
        end if
    end for
    return $\sqrt{bcRMSD^2}$
end procedure

4 CIF input/output

4.1 _atom_site

The `_atom_site` used in *rotag* as initial data is almost analogous to the one in PDBx/mmCIF [Westbrook et al., 2022] with two additional data items: `[local]_selection_state` and `[local]_selection_group`. The required data

items from `_atom_site` information for *rotag* are: `group_PDB`, `id`, `type_symbol`, `label_atom_id`, `label_alt_id`, `label_comp_id`, `label_asym_id`, `label_seq_id`, `Cartn_x`, `Cartn_y`, `Cartn_z` and `pdbx_PDB_model_num`.

```
# Example 1.
data_rotag
#
loop_
_atom_site.group_PDB
_atom_site.id
_atom_site.type_symbol
_atom_site.label_atom_id
_atom_site.label_alt_id
_atom_site.label_comp_id
_atom_site.label_asym_id
_atom_site.label_entity_id
_atom_site.label_seq_id
_atom_site.Cartn_x
_atom_site.Cartn_y
_atom_site.Cartn_z
_atom_site.pdbx_PDB_model_num
_atom_site.[local]_selection_state
_atom_site.[local]_selection_group
ATOM 999 C C . THR A 1 106 1.814 16.065 32.192 1 S ?
ATOM 1004 N N . PHE A 1 107 0.860 15.399 32.830 1 T 1
ATOM 1005 C CA . PHE A 1 107 1.093 14.763 34.096 1 T 1
ATOM 1006 C C . PHE A 1 107 0.251 13.488 34.141 1 T 1
ATOM 1007 O O . PHE A 1 107 -0.975 13.606 34.074 1 T 1
ATOM 1008 C CB . PHE A 1 107 0.674 15.777 35.213 1 T 1
ATOM 1009 C CG . PHE A 1 107 1.041 15.372 36.597 1 T 1
ATOM 1010 C CD1 . PHE A 1 107 2.310 15.659 37.086 1 T 1
ATOM 1011 C CD2 . PHE A 1 107 0.134 14.728 37.416 1 T 1
ATOM 1012 C CE1 . PHE A 1 107 2.677 15.328 38.336 1 T 1
ATOM 1013 C CE2 . PHE A 1 107 0.532 14.371 38.758 1 T 1
ATOM 1014 C CZ . PHE A 1 107 1.780 14.707 39.207 1 T 1
ATOM 1015 N N . THR A 1 108 0.888 12.330 34.303 1 S ?
```

Figure S6: **_atom_site** category with additional local data items that are essential to *rotag*.

`_atom_site.[local]_selection_state` (char)
The data item that is required for the selection of specific side-chains for the calculations. `[local]_selection_state` is in an enumerator that consists of three values: `T`, `S`, `I`. `T` signifies target atoms which rotamer library will be generated from, `S` – selected atoms that will be included in energy calculations

only and `I` – ignored atoms which coordinates will be kept but not included in the calculations.

`_atom_site.[local]_selection_group` (char)
Groups are used to select different groups of side-chains in order to compare data (dihedral angles or RMSD) or select in further analysis.

4.2 _[local]_rotamer_angle

`_[local]_rotamer_angle` category data is generated by *rotag_scan* and *rotag_library* scripts that stores data of available rotamers for further modelling with *rotag_add*.

```
# Example 2.
data_rotag
#
loop_
_[local]_rotamer_angle.id
_[local]_rotamer_angle.rotamer_id
_[local]_rotamer_angle.label_seq_id
_[local]_rotamer_angle.label_comp_id
_[local]_rotamer_angle.label_asym_id
_[local]_rotamer_angle.pdbx_PDB_model_num
_[local]_rotamer_angle.label_alt_id
_[local]_rotamer_angle.frequency
_[local]_rotamer_angle.type
_[local]_rotamer_angle.value
_[local]_rotamer_angle.units
13 7 107 PHE A 1 . 1.000000 chi1 -170.000 degrees
14 7 107 PHE A 1 . 1.000000 chi2 90.000 degrees
```

Figure S7: local **_[local]_rotamer_angle** category data.

`_[local]_rotamer_angle.id` (numb)
The unique identifier that separates every dihedral angle.

`_[local]_rotamer_angle.rotamer_id` (numb)
The identifier that separates the rotamers. The rotamer is described by the unique combination of data items: `label_seq_id`, `label_asym_id`, `pdbx_PDB_model_num` and `label_alt_id`.

`_[local]_rotamer_angle.label_seq_id` (numb)
`_[local]_rotamer_angle.label_comp_id` (char)
`_[local]_rotamer_angle.label_asym_id` (char)

`_[local]_rotamer_angle.pdbx_PDB_model_num` (num)
`_[local]_rotamer_angle.label_alt_id` (char)
The data items are analogous to PDBx/mmCIF `_atom_site` data items.

`_[local]_rotamer_angle.frequency` (numb)
Notifies the fraction of occurrences of the rotamer. Also, it could be used as probability entry. However, *rotag* does not use as probabilities only as fractions for now.

`_[local]_rotamer_angle.type` (char)
`_[local]_rotamer_angle.value` (numb)
`_[local]_rotamer_angle.units` (char)
Angle related data items. `type` stores dihedral angle name, `value` – the value of the angle and `units` – units in radians or degrees.

4.3 _[local]_rotamer_energy

Stores information on energies generated by *rotag_scan*.

```
# Example 3.
data_rotag
#
loop_
_[local]_rotamer_energy.id
_[local]_rotamer_energy.rotamer_id
_[local]_rotamer_energy.calculation_method
_[local]_rotamer_energy.value
13 13 composite 69.948321
14 14 composite 71.173040
```

Figure S8

`_[local]_rotamer_energy.id` (numb)
The unique identifier that separates rotamer energy calculations for target side-chains.

`_[local]_rotamer_energy.rotamer_id` (numb)
Identifies which rotamer was calculated with. Related to `_[local]_rotamer_angle.rotamer_id`

`_[local]_rotamer_energy.calculation_method` (char)
Identifies the method the energy was calculated. It is in enumerator form: composite (default value), hard_sphere, soft_sphere, lennard_jones, coulomb

and h_bond.

`_[local]_rotamer_energy.value` (numb)
The value of calculated potential for target side-chains in arbitrary units.

5 Basic usage cases

In order to select side-chain, the *rotag_select* has to be used:

```
$ rotag_select -t 'resid 20 && chain A' -s 'target around 5' -k 4
    dhw.cif > 4dhw-rotag-select.cif
$ rotag_scan 4dhw-rotag-select.cif > 4dhw-rotag-scan.cif
```

Then, the output of `rotag_scan` can be used either in `rotag_library` in order to filter out rotamers or in `rotag_add` with `-S` option for generating the structure.

```
$ rotag_library -M 0.1 4dhw-rotag-scan.cif > 4dhw-rotag-library.
    cif
$ rotag_add -S 4dhw-rotag-library.cif > 4dhw-rotag-add.cif
```

6 Best-case RMSD

6.1 Raw data set results

The raw analysis data of the data sets can be found in Supplementary File *rotamer-library-bc-rmsd.csv* of *SupplementaryData.zip* archive.

7 Best-case RMSD statistics

7.1 Data set 13097

Table S2: The results of paired statistics for bcRMSD comparisons between *rotag* and other libraries. * - Cohen's D ≥ 0.5 SD, ** - |Δmean| ≥ 0.1 Å, are considered to be significant.

Residue	Rotamer library	Wilcoxon's p-value	Cohen's D, SD	SD, Å	Δmean, Å	95% CI, Å	N
ARG	Dunbrack (BBDEP)	1.281e-5	0.45	0.694	0.312	[0.254; 0.371]	919
ARG	Dynameomics (BBDEP)	0.04459	0.393	0.696	0.274	[0.215; 0.332]	919
ARG	Ultimate (BBIND)	0.001227	0.42	0.692	0.291	[0.233; 0.349]	933
ARG	Dynameomics (BBIND)	0.1964	0.373	0.693	0.258	[0.201; 0.316]	933
ASP	Dunbrack (BBDEP)	7.233e-52	0.589 *	0.418	0.246 **	[0.21; 0.282]	914
ASP	Dynameomics (BBDEP)	0.009186	0.29	0.407	0.118	[0.08; 0.156]	914
ASP	Ultimate (BBIND)	0.002745	0.249	0.425	0.106	[0.07; 0.142]	940
ASP	Dynameomics (BBIND)	0.02638	0.276	0.409	0.113	[0.076; 0.15]	940
ASN	Dunbrack (BBDEP)	1.473e-13	0.385	0.472	0.182	[0.144; 0.219]	923
ASN	Dynameomics (BBDEP)	0.1875	0.278	0.463	0.129	[0.091; 0.166]	923
ASN	Ultimate (BBIND)	8.251e-20	-0.116	0.495	-0.057	[-0.096; -0.018]	939
ASN	Dynameomics (BBIND)	0.453	0.208	0.479	0.1	[0.062; 0.137]	939
CYS	Dunbrack (BBDEP)	6.46e-26	0.018	0.374	0.007	[-0.027; 0.04]	724
CYS	Dynameomics (BBDEP)	7.699e-47	-0.085	0.369	-0.032	[-0.066; 0.003]	724
CYS	Ultimate (BBIND)	1.091e-39	-0.044	0.363	-0.016	[-0.048; 0.016]	772
CYS	Dynameomics (BBIND)	1.715e-51	-0.131	0.36	-0.047	[-0.08; -0.014]	772
GLU	Dunbrack (BBDEP)	0.9067	0.291	0.439	0.128	[0.091; 0.165]	893
GLU	Dynameomics (BBDEP)	4.387e-10	0.116	0.442	0.051	[0.015; 0.088]	893
GLU	Ultimate (BBIND)	4.262e-13	0.01	0.435	0.004	[-0.034; 0.043]	918
GLU	Dynameomics (BBIND)	1.494e-13	0.065	0.445	0.029	[-0.006; 0.064]	918
GLN	Dunbrack (BBDEP)	6.65e-19	-0.136	0.317	-0.043	[-0.067; -0.019]	902
GLN	Dynameomics (BBDEP)	1.355e-46	-0.305	0.314	-0.096	[-0.119; -0.073]	902
GLN	Ultimate (BBIND)	1.606e-76	-0.729 *	0.357	-0.26 **	[-0.29; -0.23]	914
GLN	Dynameomics (BBIND)	4.413e-55	-0.331	0.332	-0.11	[-0.134; -0.086]	914
HIS	Dunbrack (BBDEP)	2.152e-24	-0.073	0.455	-0.033	[-0.07; 0.003]	875
HIS	Dynameomics (BBDEP)	2.356e-37	-0.115	0.448	-0.051	[-0.088; -0.015]	875
HIS	Ultimate (BBIND)	1.705e-57	-0.235	0.462	-0.109	[-0.145; -0.072]	919
HIS	Dynameomics (BBIND)	1.317e-45	-0.14	0.458	-0.064	[-0.1; -0.029]	919
ILE	Dunbrack (BBDEP)	0.04075	0.217	0.206	0.045	[0.028; 0.061]	918
ILE	Dynameomics (BBDEP)	8.885e-20	0.031	0.206	0.006	[-0.011; 0.024]	918
ILE	Ultimate (BBIND)	5.258e-9	0.096	0.214	0.021	[0.004; 0.037]	927
ILE	Dynameomics (BBIND)	4.778e-22	-0.006	0.212	-0.001	[-0.019; 0.016]	927
LEU	Dunbrack (BBDEP)	3.111e-77	-0.575	0.161	-0.092	[-0.105; -0.08]	956
LEU	Dynameomics (BBDEP)	3.175e-121	-0.827 *	0.17	-0.14 **	[-0.153; -0.128]	956
LEU	Ultimate (BBIND)	1.013e-113	-0.75 *	0.17	-0.127 **	[-0.139; -0.115]	963
LEU	Dynameomics (BBIND)	2.026e-132	-0.919 *	0.177	-0.163 **	[-0.175; -0.15]	963
LYS	Dunbrack (BBDEP)	2.502e-16	-0.284	0.227	-0.064	[-0.084; -0.045]	891
LYS	Dynameomics (BBDEP)	4.79e-32	-0.414	0.229	-0.095	[-0.114; -0.076]	891
LYS	Ultimate (BBIND)	6.978e-25	-0.329	0.243	-0.08	[-0.101; -0.059]	907
LYS	Dynameomics (BBIND)	5.305e-41	-0.415	0.241	-0.1	[-0.12; -0.08]	907
MET	Dunbrack (BBDEP)	1.292e-62	-0.912 *	0.182	-0.166 **	[-0.183; -0.149]	670
MET	Dynameomics (BBDEP)	4.09e-85	-1.11 *	0.203	-0.225 **	[-0.243; -0.207]	670
MET	Ultimate (BBIND)	3.629e-79	-0.924 *	0.209	-0.193 **	[-0.211; -0.176]	743
MET	Dynameomics (BBIND)	1.821e-100	-1.124 *	0.214	-0.24 **	[-0.259; -0.222]	743
PHE	Dunbrack (BBDEP)	2.27e-39	-0.336	0.318	-0.107	[-0.134; -0.08]	951
PHE	Dynameomics (BBDEP)	8.829e-91	-0.62 *	0.325	-0.201 **	[-0.229; -0.174]	951
PHE	Ultimate (BBIND)	6.13e-88	-0.602 *	0.328	-0.197 **	[-0.225; -0.17]	958

PHE	Dynameomics (BBIND)	1.493e-100	-0.695 *	0.328	-0.228 **	[-0.256; -0.201]	958
SER	Dunbrack (BBDEP)	2.153e-14	0.164	0.384	0.063	[0.031; 0.095]	874
SER	Dynameomics (BBDEP)	2.448e-77	-0.453	0.405	-0.183	[-0.216; -0.151]	874
SER	Ultimate (BBIND)	5.14e-20	0.137	0.386	0.053	[0.022; 0.084]	912
SER	Dynameomics (BBIND)	4.843e-85	-0.509 *	0.403	-0.205 **	[-0.236; -0.174]	912
TRP	Dunbrack (BBDEP)	1.573e-26	-0.059	0.638	-0.037	[-0.094; 0.019]	876
TRP	Dynameomics (BBDEP)	1.427e-59	-0.316	0.671	-0.212	[-0.269; -0.155]	876
TRP	Ultimate (BBIND)	8.172e-74	-0.414	0.674	-0.279	[-0.338; -0.22]	882
TRP	Dynameomics (BBIND)	4.345e-68	-0.371	0.668	-0.248	[-0.305; -0.192]	882
THR	Dunbrack (BBDEP)	0.02667	-0.077	0.127	-0.01	[-0.019; 0]	938
THR	Dynameomics (BBDEP)	2.133e-148	-1.831 *	0.19	-0.348 **	[-0.362; -0.334]	938
THR	Ultimate (BBIND)	6.094e-9	-0.133	0.133	-0.018	[-0.027; -0.008]	955
THR	Dynameomics (BBIND)	7.366e-153	-2.045 *	0.184	-0.377 **	[-0.39; -0.363]	955
TYR	Dunbrack (BBDEP)	2.34e-49	-0.284	0.449	-0.128	[-0.165; -0.09]	948
TYR	Dynameomics (BBDEP)	2.863e-83	-0.453	0.456	-0.207	[-0.244; -0.169]	948
TYR	Ultimate (BBIND)	2.174e-76	-0.384	0.492	-0.189	[-0.229; -0.149]	956
TYR	Dynameomics (BBIND)	3.063e-85	-0.444	0.495	-0.22	[-0.259; -0.181]	956
VAL	Dunbrack (BBDEP)	2.662e-18	0.309	0.11	0.034	[0.026; 0.042]	948
VAL	Dynameomics (BBDEP)	1.787e-10	-0.256	0.124	-0.032	[-0.041; -0.023]	948
VAL	Ultimate (BBIND)	2.905e-4	0.13	0.113	0.015	[0.007; 0.023]	954
VAL	Dynameomics (BBIND)	2.817e-10	-0.316	0.123	-0.039	[-0.045; -0.032]	954

7.2 Data set 16226

Table S3: The results of paired statistics for bcRMSD comparisons between *rotag* and other libraries. * - Cohen's D ≥ 0.5 SD, ** - |Δmean| ≥ 0.1 Å, are considered to be significant.

Residue	Rotamer library	Wilcoxon's p-value	Cohen's D, SD	SD, Å	Δmean, Å	95% CI, Å	N
ARG	Dunbrack (BBDEP)	4.045e-6	0.444	0.719	0.319	[0.258; 0.38]	896
ARG	Dynameomics (BBDEP)	0.02857	0.385	0.718	0.276	[0.215; 0.337]	896
ARG	Ultimate (BBIND)	3.836e-4	0.418	0.723	0.302	[0.242; 0.363]	900
ARG	Dynameomics (BBIND)	0.1254	0.38	0.717	0.272	[0.212; 0.333]	900
ASP	Dunbrack (BBDEP)	1.94e-36	0.549 *	0.466	0.256 **	[0.216; 0.296]	926
ASP	Dynameomics (BBDEP)	0.42	0.291	0.453	0.132	[0.089; 0.175]	926
ASP	Ultimate (BBIND)	0.07514	0.275	0.472	0.13	[0.088; 0.171]	943
ASP	Dynameomics (BBIND)	0.8131	0.284	0.458	0.13	[0.089; 0.172]	943
ASN	Dunbrack (BBDEP)	3.67e-10	0.369	0.416	0.153	[0.119; 0.187]	928
ASN	Dynameomics (BBDEP)	0.8688	0.221	0.41	0.091	[0.056; 0.125]	928
ASN	Ultimate (BBIND)	1.721e-26	-0.219	0.433	-0.095	[-0.13; -0.059]	934
ASN	Dynameomics (BBIND)	0.00496	0.136	0.41	0.056	[0.021; 0.09]	934
CYS	Dunbrack (BBDEP)	8.373e-11	0.209	0.514	0.107	[0.06; 0.155]	697
CYS	Dynameomics (BBDEP)	1.102e-21	0.144	0.502	0.072	[0.024; 0.121]	697
CYS	Ultimate (BBIND)	8.17e-23	0.161	0.496	0.08	[0.035; 0.125]	744
CYS	Dynameomics (BBIND)	3.364e-33	0.092	0.487	0.045	[-0.001; 0.091]	744
GLU	Dunbrack (BBDEP)	0.4345	0.236	0.401	0.095	[0.063; 0.127]	895
GLU	Dynameomics (BBDEP)	3.432e-14	0.036	0.398	0.014	[-0.018; 0.047]	895
GLU	Ultimate (BBIND)	1.067e-16	-0.061	0.406	-0.025	[-0.06; 0.01]	912
GLU	Dynameomics (BBIND)	5.486e-19	-0.026	0.409	-0.011	[-0.043; 0.021]	912
GLN	Dunbrack (BBDEP)	2.381e-18	-0.092	0.335	-0.031	[-0.058; -0.004]	905
GLN	Dynameomics (BBDEP)	3.44e-49	-0.27	0.329	-0.089	[-0.116; -0.062]	905
GLN	Ultimate (BBIND)	1.573e-79	-0.71 *	0.358	-0.254 **	[-0.286; -0.221]	909
GLN	Dynameomics (BBIND)	1.306e-55	-0.313	0.334	-0.105	[-0.131; -0.078]	909
HIS	Dunbrack (BBDEP)	6.199e-23	-0.057	0.446	-0.025	[-0.062; 0.012]	858
HIS	Dynameomics (BBDEP)	1.278e-32	-0.11	0.442	-0.049	[-0.086; -0.012]	858
HIS	Ultimate (BBIND)	3.141e-56	-0.262	0.454	-0.119	[-0.156; -0.083]	912
HIS	Dynameomics (BBIND)	2.759e-47	-0.164	0.445	-0.073	[-0.109; -0.037]	912
ILE	Dunbrack (BBDEP)	9.859e-7	0.13	0.199	0.026	[0.01; 0.042]	914
ILE	Dynameomics (BBDEP)	2.602e-28	-0.028	0.2	-0.006	[-0.022; 0.011]	914
ILE	Ultimate (BBIND)	5.309e-17	0.004	0.202	0.001	[-0.015; 0.017]	918
ILE	Dynameomics (BBIND)	2.367e-30	-0.087	0.202	-0.018	[-0.034; -0.001]	918
LEU	Dunbrack (BBDEP)	1.871e-79	-0.531	0.181	-0.096	[-0.111; -0.081]	949
LEU	Dynameomics (BBDEP)	2.394e-120	-0.761 *	0.187	-0.142 **	[-0.158; -0.127]	949
LEU	Ultimate (BBIND)	1.118e-108	-0.686 *	0.182	-0.125 **	[-0.14; -0.11]	955
LEU	Dynameomics (BBIND)	9.664e-124	-0.828 *	0.194	-0.161 **	[-0.177; -0.144]	955
LYS	Dunbrack (BBDEP)	9.199e-24	-0.333	0.236	-0.079	[-0.098; -0.059]	890
LYS	Dynameomics (BBDEP)	2.232e-39	-0.44	0.235	-0.104	[-0.123; -0.084]	890
LYS	Ultimate (BBIND)	5.743e-28	-0.345	0.249	-0.086	[-0.106; -0.065]	899
LYS	Dynameomics (BBIND)	4.141e-48	-0.44	0.248	-0.109	[-0.129; -0.089]	899
MET	Dunbrack (BBDEP)	1.922e-73	-0.914 *	0.201	-0.183 **	[-0.201; -0.166]	696
MET	Dynameomics (BBDEP)	1.017e-93	-1.168 *	0.204	-0.238 **	[-0.255; -0.221]	696
MET	Ultimate (BBIND)	3.395e-87	-1.011 *	0.207	-0.21 **	[-0.227; -0.192]	734
MET	Dynameomics (BBIND)	2.157e-98	-1.159 *	0.214	-0.248 **	[-0.265; -0.231]	734
PHE	Dunbrack (BBDEP)	7.366e-45	-0.328	0.327	-0.107	[-0.137; -0.078]	945
PHE	Dynameomics (BBDEP)	3.522e-89	-0.589 *	0.337	-0.199 **	[-0.228; -0.169]	945
PHE	Ultimate (BBIND)	1.768e-87	-0.561 *	0.344	-0.193 **	[-0.223; -0.162]	953
PHE	Dynameomics (BBIND)	5.772e-103	-0.669 *	0.341	-0.228 **	[-0.259; -0.198]	953
SER	Dunbrack (BBDEP)	1.334e-9	0.245	0.462	0.113	[0.076; 0.151]	893

SER	Dynameomics (BBDEP)	1.896e-60	-0.293	0.484	-0.142	[-0.178; -0.105]	893
SER	Ultimate (BBIND)	1.832e-11	0.228	0.459	0.104	[0.067; 0.142]	904
SER	Dynameomics (BBIND)	1.155e-63	-0.355	0.481	-0.171	[-0.207; -0.135]	904
TRP	Dunbrack (BBDEP)	2.606e-23	-0.075	0.593	-0.045	[-0.098; 0.009]	851
TRP	Dynameomics (BBDEP)	6.916e-55	-0.358	0.617	-0.221	[-0.277; -0.165]	851
TRP	Ultimate (BBIND)	4.948e-65	-0.438	0.623	-0.273	[-0.328; -0.218]	855
TRP	Dynameomics (BBIND)	1.919e-60	-0.4	0.616	-0.246	[-0.301; -0.192]	855
THR	Dunbrack (BBDEP)	0.004831	-0.183	0.101	-0.019	[-0.026; -0.011]	934
THR	Dynameomics (BBDEP)	1.891e-148	-1.858 *	0.191	-0.355 **	[-0.369; -0.341]	934
THR	Ultimate (BBIND)	4.489e-11	-0.262	0.104	-0.027	[-0.035; -0.02]	940
THR	Dynameomics (BBIND)	3.949e-154	-2.147 *	0.181	-0.388 **	[-0.4; -0.376]	940
TYR	Dunbrack (BBDEP)	8.135e-45	-0.179	0.549	-0.098	[-0.146; -0.051]	937
TYR	Dynameomics (BBDEP)	1.202e-78	-0.309	0.549	-0.17	[-0.218; -0.122]	937
TYR	Ultimate (BBIND)	3.24e-78	-0.327	0.554	-0.181	[-0.229; -0.134]	941
TYR	Dynameomics (BBIND)	3.89e-92	-0.381	0.552	-0.21	[-0.257; -0.162]	941
VAL	Dunbrack (BBDEP)	1.034e-13	0.264	0.102	0.027	[0.02; 0.034]	946
VAL	Dynameomics (BBDEP)	7.72e-17	-0.335	0.122	-0.041	[-0.049; -0.032]	946
VAL	Ultimate (BBIND)	0.01583	0.081	0.105	0.009	[0.001; 0.016]	948
VAL	Dynameomics (BBIND)	3.314e-15	-0.353	0.118	-0.042	[-0.048; -0.036]	948

7.3 Data set 15222

Table S4: The results of paired statistics for bcRMSD comparisons between *rotag* and other libraries. * - Cohen's D ≥ 0.5 SD, ** - |Δmean| ≥ 0.1 Å, are considered to be significant.

Residue	Rotamer library	Wilcoxon's p-value	Cohen's D, SD	SD, Å	Δmean, Å	95% CI, Å	N
ARG	Dunbrack (BBDEP)	3.204e-9	0.488	0.763	0.372	[0.307; 0.436]	895
ARG	Dynameomics (BBDEP)	1.07e-4	0.434	0.765	0.332	[0.267; 0.396]	895
ARG	Ultimate (BBIND)	2.829e-6	0.456	0.763	0.348	[0.285; 0.412]	908
ARG	Dynameomics (BBIND)	0.001639	0.418	0.764	0.32	[0.256; 0.383]	908
ASP	Dunbrack (BBDEP)	1.997e-39	0.566 *	0.438	0.248 **	[0.209; 0.287]	905
ASP	Dynameomics (BBDEP)	0.09932	0.272	0.431	0.117	[0.077; 0.158]	905
ASP	Ultimate (BBIND)	0.1017	0.242	0.446	0.108	[0.069; 0.147]	930
ASP	Dynameomics (BBIND)	0.4709	0.255	0.433	0.11	[0.071; 0.15]	930
ASN	Dunbrack (BBDEP)	1.411e-11	0.386	0.434	0.168	[0.132; 0.204]	910
ASN	Dynameomics (BBDEP)	0.709	0.249	0.428	0.107	[0.07; 0.143]	910
ASN	Ultimate (BBIND)	1.412e-21	-0.145	0.46	-0.067	[-0.104; -0.029]	931
ASN	Dynameomics (BBIND)	0.2192	0.2	0.433	0.087	[0.049; 0.124]	931
CYS	Dunbrack (BBDEP)	4.278e-18	0.1	0.426	0.043	[0.005; 0.08]	708
CYS	Dynameomics (BBDEP)	1.431e-32	0.005	0.42	0.002	[-0.036; 0.04]	708
CYS	Ultimate (BBIND)	9.164e-30	0.047	0.416	0.019	[-0.016; 0.055]	759
CYS	Dynameomics (BBIND)	4.897e-48	-0.065	0.41	-0.026	[-0.063; 0.01]	759
GLU	Dunbrack (BBDEP)	0.9851	0.272	0.442	0.12	[0.085; 0.156]	896
GLU	Dynameomics (BBDEP)	1.277e-13	0.088	0.438	0.039	[0.002; 0.075]	896
GLU	Ultimate (BBIND)	2.571e-13	0	0.445	0	[-0.038; 0.038]	918
GLU	Dynameomics (BBIND)	4.94e-16	0.07	0.447	0.031	[-0.004; 0.067]	918
GLN	Dunbrack (BBDEP)	7.305e-17	-0.048	0.368	-0.018	[-0.047; 0.012]	901
GLN	Dynameomics (BBDEP)	1.099e-45	-0.219	0.364	-0.08	[-0.108; -0.051]	901
GLN	Ultimate (BBIND)	2.871e-69	-0.622 *	0.386	-0.24 **	[-0.274; -0.207]	916
GLN	Dynameomics (BBIND)	1.526e-50	-0.258	0.37	-0.095	[-0.124; -0.067]	916
HIS	Dunbrack (BBDEP)	1.276e-15	0.008	0.495	0.004	[-0.038; 0.046]	839
HIS	Dynameomics (BBDEP)	1.733e-20	-0.012	0.488	-0.006	[-0.048; 0.036]	839
HIS	Ultimate (BBIND)	6.575e-46	-0.175	0.503	-0.088	[-0.128; -0.047]	898
HIS	Dynameomics (BBIND)	5.257e-32	-0.067	0.495	-0.033	[-0.073; 0.007]	898
ILE	Dunbrack (BBDEP)	9.246e-6	0.13	0.195	0.026	[0.009; 0.042]	910
ILE	Dynameomics (BBDEP)	7.057e-26	-0.039	0.199	-0.008	[-0.025; 0.009]	910
ILE	Ultimate (BBIND)	1.68e-15	0.011	0.203	0.002	[-0.014; 0.019]	922
ILE	Dynameomics (BBIND)	4.404e-29	-0.077	0.202	-0.016	[-0.033; 0.001]	922
LEU	Dunbrack (BBDEP)	6.433e-90	-0.707 *	0.163	-0.115 **	[-0.126; -0.105]	933
LEU	Dynameomics (BBDEP)	2.128e-127	-0.95 *	0.166	-0.158 **	[-0.168; -0.148]	933
LEU	Ultimate (BBIND)	3.421e-123	-0.833 *	0.176	-0.146 **	[-0.156; -0.136]	945
LEU	Dynameomics (BBIND)	2.173e-137	-1.002 *	0.18	-0.18 **	[-0.191; -0.169]	945
LYS	Dunbrack (BBDEP)	1.454e-23	-0.333	0.23	-0.076	[-0.095; -0.058]	887
LYS	Dynameomics (BBDEP)	7.079e-35	-0.447	0.234	-0.104	[-0.123; -0.086]	887
LYS	Ultimate (BBIND)	4.166e-33	-0.415	0.236	-0.098	[-0.117; -0.079]	903
LYS	Dynameomics (BBIND)	4.976e-49	-0.516 *	0.239	-0.123 **	[-0.141; -0.105]	903
MET	Dunbrack (BBDEP)	9.824e-75	-0.957 *	0.196	-0.188 **	[-0.204; -0.171]	708
MET	Dynameomics (BBDEP)	1.039e-95	-1.091 *	0.228	-0.249 **	[-0.267; -0.231]	708
MET	Ultimate (BBIND)	1.824e-85	-0.988 *	0.21	-0.207 **	[-0.224; -0.191]	748
MET	Dynameomics (BBIND)	3.638e-101	-1.19 *	0.216	-0.256 **	[-0.274; -0.239]	748
PHE	Dunbrack (BBDEP)	1.657e-36	-0.228	0.394	-0.09	[-0.123; -0.056]	941
PHE	Dynameomics (BBDEP)	4.41e-84	-0.458	0.4	-0.183	[-0.217; -0.149]	941
PHE	Ultimate (BBIND)	1.802e-82	-0.462	0.403	-0.187	[-0.22; -0.153]	951
PHE	Dynameomics (BBIND)	8.987e-101	-0.547 *	0.4	-0.219 **	[-0.252; -0.185]	951
SER	Dunbrack (BBDEP)	1.497e-13	0.187	0.409	0.076	[0.041; 0.111]	860

SER	Dynameomics (BBDEP)	1.285e-69	-0.407	0.439	-0.179	[-0.213; -0.145]	860
SER	Ultimate (BBIND)	1.534e-16	0.166	0.404	0.067	[0.033; 0.101]	898
SER	Dynameomics (BBIND)	5.543e-76	-0.449	0.431	-0.193	[-0.226; -0.16]	898
TRP	Dunbrack (BBDEP)	3.032e-25	-0.031	0.642	-0.02	[-0.077; 0.038]	839
TRP	Dynameomics (BBDEP)	5.431e-56	-0.261	0.665	-0.174	[-0.231; -0.116]	839
TRP	Ultimate (BBIND)	1.241e-68	-0.35	0.674	-0.235	[-0.295; -0.176]	844
TRP	Dynameomics (BBIND)	3.244e-64	-0.314	0.664	-0.209	[-0.266; -0.151]	844
THR	Dunbrack (BBDEP)	0.004902	-0.1	0.13	-0.013	[-0.021; -0.004]	927
THR	Dynameomics (BBDEP)	6.82e-144	-1.743 *	0.19	-0.331 **	[-0.346; -0.317]	927
THR	Ultimate (BBIND)	1.121e-7	-0.13	0.154	-0.02	[-0.029; -0.011]	946
THR	Dynameomics (BBIND)	9.155e-151	-1.937 *	0.195	-0.377 **	[-0.391; -0.364]	946
TYR	Dunbrack (BBDEP)	5.946e-45	-0.232	0.468	-0.109	[-0.149; -0.069]	932
TYR	Dynameomics (BBDEP)	1.933e-77	-0.389	0.471	-0.183	[-0.223; -0.144]	932
TYR	Ultimate (BBIND)	4.737e-77	-0.393	0.476	-0.187	[-0.227; -0.147]	947
TYR	Dynameomics (BBIND)	4.03e-87	-0.452	0.476	-0.215	[-0.255; -0.175]	947
VAL	Dunbrack (BBDEP)	8.901e-14	0.279	0.109	0.03	[0.023; 0.038]	942
VAL	Dynameomics (BBDEP)	6.144e-12	-0.26	0.127	-0.033	[-0.042; -0.024]	942
VAL	Ultimate (BBIND)	0.09545	0.067	0.116	0.008	[0; 0.016]	957
VAL	Dynameomics (BBIND)	4.199e-15	-0.319	0.131	-0.042	[-0.048; -0.035]	957

7.4 Data set 16089

Table S5: The results of paired statistics for bcRMSD comparisons between *rotag* and other libraries. * - Cohen's D $\geq$ 0.5 SD, ** - $|\Delta$mean$|$ $\geq$ 0.1 Å, are considered to be significant.

Residue	Rotamer library	Wilcoxon's p-value	Cohen's D, SD	SD, Å	Δmean, Å	95% CI, Å	N
ARG	Dunbrack (BBDEP)	9.482e-7	0.473	0.738	0.349	[0.285; 0.414]	892
ARG	Dynameomics (BBDEP)	0.0482	0.408	0.736	0.3	[0.236; 0.365]	892
ARG	Ultimate (BBIND)	2.395e-4	0.434	0.746	0.324	[0.261; 0.387]	914
ARG	Dynameomics (BBIND)	0.1031	0.391	0.745	0.292	[0.228; 0.355]	914
ASP	Dunbrack (BBDEP)	7.154e-35	0.521 *	0.432	0.225 **	[0.188; 0.263]	889
ASP	Dynameomics (BBDEP)	0.8376	0.222	0.425	0.094	[0.055; 0.134]	889
ASP	Ultimate (BBIND)	0.3313	0.21	0.443	0.093	[0.055; 0.131]	927
ASP	Dynameomics (BBIND)	0.5608	0.216	0.432	0.093	[0.055; 0.131]	927
ASN	Dunbrack (BBDEP)	8.621e-11	0.38	0.419	0.159	[0.124; 0.195]	901
ASN	Dynameomics (BBDEP)	0.7288	0.221	0.414	0.091	[0.056; 0.127]	901
ASN	Ultimate (BBIND)	1.777e-25	-0.205	0.443	-0.091	[-0.127; -0.054]	929
ASN	Dynameomics (BBIND)	0.008495	0.153	0.416	0.064	[0.028; 0.099]	929
CYS	Dunbrack (BBDEP)	5.197e-17	0.121	0.442	0.054	[0.013; 0.095]	720
CYS	Dynameomics (BBDEP)	7.504e-35	0.039	0.44	0.017	[-0.024; 0.059]	720
CYS	Ultimate (BBIND)	3.766e-25	0.074	0.43	0.032	[-0.007; 0.071]	761
CYS	Dynameomics (BBIND)	3.638e-41	-0.007	0.425	-0.003	[-0.043; 0.037]	761
GLU	Dunbrack (BBDEP)	0.263	0.252	0.438	0.11	[0.073; 0.147]	880
GLU	Dynameomics (BBDEP)	1.86e-12	0.09	0.442	0.04	[0.004; 0.075]	880
GLU	Ultimate (BBIND)	5.942e-15	-0.023	0.447	-0.01	[-0.049; 0.029]	909
GLU	Dynameomics (BBIND)	2.124e-13	0.059	0.451	0.027	[-0.008; 0.062]	909
GLN	Dunbrack (BBDEP)	3.346e-25	-0.151	0.342	-0.052	[-0.079; -0.025]	895
GLN	Dynameomics (BBDEP)	2.08e-54	-0.31	0.34	-0.105	[-0.132; -0.079]	895
GLN	Ultimate (BBIND)	2.032e-82	-0.753 *	0.366	-0.276 **	[-0.307; -0.244]	915
GLN	Dynameomics (BBIND)	5.733e-63	-0.372	0.343	-0.127	[-0.154; -0.101]	915
HIS	Dunbrack (BBDEP)	2.855e-19	-0.019	0.458	-0.009	[-0.048; 0.03]	847
HIS	Dynameomics (BBDEP)	1.024e-23	-0.036	0.45	-0.016	[-0.056; 0.023]	847
HIS	Ultimate (BBIND)	2.743e-49	-0.177	0.478	-0.084	[-0.123; -0.046]	915
HIS	Dynameomics (BBIND)	1.643e-33	-0.061	0.469	-0.029	[-0.067; 0.01]	915
ILE	Dunbrack (BBDEP)	3.916e-6	0.178	0.199	0.035	[0.019; 0.052]	913
ILE	Dynameomics (BBDEP)	1.099e-23	0.006	0.201	0.001	[-0.016; 0.019]	913
ILE	Ultimate (BBIND)	1.956e-13	0.059	0.203	0.012	[-0.005; 0.029]	928
ILE	Dynameomics (BBIND)	4.489e-26	-0.049	0.204	-0.01	[-0.028; 0.008]	928
LEU	Dunbrack (BBDEP)	3.489e-76	-0.616	0.151	-0.093	[-0.103; -0.082]	944
LEU	Dynameomics (BBDEP)	5.007e-119	-0.896 *	0.158	-0.141 **	[-0.152; -0.13]	944
LEU	Ultimate (BBIND)	1.241e-112	-0.805 *	0.166	-0.134 **	[-0.145; -0.122]	955
LEU	Dynameomics (BBIND)	3.55e-129	-0.981 *	0.169	-0.166 **	[-0.178; -0.154]	955
LYS	Dunbrack (BBDEP)	3.896e-24	-0.332	0.241	-0.08	[-0.099; -0.061]	860
LYS	Dynameomics (BBDEP)	6.255e-38	-0.44	0.245	-0.108	[-0.126; -0.089]	860
LYS	Ultimate (BBIND)	7.732e-34	-0.394	0.252	-0.099	[-0.118; -0.08]	890
LYS	Dynameomics (BBIND)	4.457e-52	-0.491	0.248	-0.122	[-0.14; -0.104]	890
MET	Dunbrack (BBDEP)	1.061e-74	-0.941 *	0.193	-0.181 **	[-0.198; -0.165]	738
MET	Dynameomics (BBDEP)	9.424e-98	-1.175 *	0.2	-0.235 **	[-0.251; -0.218]	738
MET	Ultimate (BBIND)	4.281e-85	-1.026 *	0.196	-0.201 **	[-0.217; -0.184]	764
MET	Dynameomics (BBIND)	8.135e-98	-1.149 *	0.207	-0.237 **	[-0.254; -0.22]	764
PHE	Dunbrack (BBDEP)	4.663e-38	-0.336	0.31	-0.104	[-0.13; -0.078]	937
PHE	Dynameomics (BBDEP)	7.995e-89	-0.637 *	0.316	-0.201 **	[-0.228; -0.175]	937
PHE	Ultimate (BBIND)	2.673e-90	-0.649 *	0.322	-0.209 **	[-0.235; -0.182]	949
PHE	Dynameomics (BBIND)	1.407e-104	-0.756 *	0.317	-0.239 **	[-0.266; -0.212]	949
SER	Dunbrack (BBDEP)	7.189e-16	0.214	0.442	0.094	[0.058; 0.131]	862

SER	Dynameomics (BBDEP)	2.934e-63	-0.352	0.465	-0.164	[-0.2; -0.127]	862
SER	Ultimate (BBIND)	1.958e-19	0.186	0.439	0.082	[0.046; 0.118]	898
SER	Dynameomics (BBIND)	2.814e-69	-0.408	0.457	-0.186	[-0.222; -0.151]	898
TRP	Dunbrack (BBDEP)	1.437e-24	-0.059	0.593	-0.035	[-0.087; 0.016]	852
TRP	Dynameomics (BBDEP)	3.077e-58	-0.34	0.616	-0.209	[-0.263; -0.156]	852
TRP	Ultimate (BBIND)	2.451e-75	-0.411	0.646	-0.265	[-0.321; -0.21]	858
TRP	Dynameomics (BBIND)	8.978e-68	-0.371	0.638	-0.237	[-0.291; -0.182]	858
THR	Dunbrack (BBDEP)	0.002389	-0.097	0.13	-0.013	[-0.023; -0.002]	911
THR	Dynameomics (BBDEP)	3.09e-143	-1.849 *	0.187	-0.346 **	[-0.361; -0.332]	911
THR	Ultimate (BBIND)	2.961e-8	-0.152	0.138	-0.021	[-0.031; -0.011]	937
THR	Dynameomics (BBIND)	1.904e-150	-2.123 *	0.183	-0.388 **	[-0.401; -0.374]	937
TYR	Dunbrack (BBDEP)	1.001e-42	-0.337	0.381	-0.129	[-0.159; -0.098]	928
TYR	Dynameomics (BBDEP)	4.479e-74	-0.507 *	0.384	-0.194 **	[-0.226; -0.163]	928
TYR	Ultimate (BBIND)	1.013e-74	-0.525 *	0.39	-0.205 **	[-0.235; -0.174]	937
TYR	Dynameomics (BBIND)	1.033e-83	-0.587 *	0.39	-0.229 **	[-0.26; -0.198]	937
VAL	Dunbrack (BBDEP)	1.15e-10	0.217	0.111	0.024	[0.016; 0.032]	931
VAL	Dynameomics (BBDEP)	6.414e-19	-0.371	0.132	-0.049	[-0.058; -0.039]	931
VAL	Ultimate (BBIND)	0.06382	0.051	0.114	0.006	[-0.002; 0.013]	944
VAL	Dynameomics (BBIND)	2.238e-15	-0.377	0.131	-0.05	[-0.056; -0.043]	944

7.5 Data set 16931

Table S6: The results of paired statistics for bcRMSD comparisons between *rotag* and other libraries. * - Cohen's D ≥ 0.5 SD, ** - |Δmean| ≥ 0.1 Å, are considered to be significant.

Residue	Rotamer library	Wilcoxon's p-value	Cohen's D, SD	SD, Å	Δmean, Å	95% CI, Å	N
ARG	Dunbrack (BBDEP)	5.271e-9	0.481	0.777	0.374	[0.307; 0.44]	903
ARG	Dynameomics (BBDEP)	0.001709	0.414	0.782	0.324	[0.258; 0.39]	903
ARG	Ultimate (BBIND)	2.141e-7	0.457	0.782	0.358	[0.291; 0.424]	904
ARG	Dynameomics (BBIND)	0.005826	0.415	0.775	0.322	[0.255; 0.388]	904
ASP	Dunbrack (BBDEP)	8.655e-37	0.55 *	0.462	0.254 **	[0.213; 0.295]	896
ASP	Dynameomics (BBDEP)	0.6442	0.273	0.455	0.124	[0.082; 0.167]	896
ASP	Ultimate (BBIND)	0.03827	0.286	0.465	0.133	[0.092; 0.175]	929
ASP	Dynameomics (BBIND)	0.692	0.273	0.454	0.124	[0.082; 0.166]	929
ASN	Dunbrack (BBDEP)	1.055e-11	0.411	0.463	0.19	[0.151; 0.23]	927
ASN	Dynameomics (BBDEP)	0.802	0.27	0.464	0.125	[0.086; 0.165]	927
ASN	Ultimate (BBIND)	6.516e-22	-0.119	0.49	-0.058	[-0.097; -0.019]	936
ASN	Dynameomics (BBIND)	0.0179	0.187	0.467	0.087	[0.049; 0.126]	936
CYS	Dunbrack (BBDEP)	1.928e-11	0.172	0.469	0.081	[0.038; 0.124]	719
CYS	Dynameomics (BBDEP)	2.69e-25	0.084	0.464	0.039	[-0.005; 0.083]	719
CYS	Ultimate (BBIND)	9.588e-23	0.121	0.451	0.055	[0.014; 0.096]	763
CYS	Dynameomics (BBIND)	1.353e-32	0.043	0.45	0.02	[-0.022; 0.061]	763
GLU	Dunbrack (BBDEP)	0.1855	0.293	0.421	0.124	[0.089; 0.159]	888
GLU	Dynameomics (BBDEP)	4.829e-11	0.086	0.424	0.036	[0.001; 0.071]	888
GLU	Ultimate (BBIND)	4.748e-12	-0.014	0.425	-0.006	[-0.044; 0.032]	903
GLU	Dynameomics (BBIND)	1.154e-14	0.041	0.431	0.018	[-0.017; 0.052]	903
GLN	Dunbrack (BBDEP)	7.316e-19	-0.102	0.312	-0.032	[-0.058; -0.006]	910
GLN	Dynameomics (BBDEP)	2.11e-51	-0.327	0.31	-0.101	[-0.126; -0.076]	910
GLN	Ultimate (BBIND)	1.746e-77	-0.799 *	0.346	-0.276 **	[-0.307; -0.245]	918
GLN	Dynameomics (BBIND)	3.586e-59	-0.399	0.314	-0.125	[-0.15; -0.1]	918
HIS	Dunbrack (BBDEP)	4.451e-18	0.035	0.564	0.02	[-0.027; 0.066]	866
HIS	Dynameomics (BBDEP)	2.975e-23	0.015	0.573	0.008	[-0.037; 0.053]	866
HIS	Ultimate (BBIND)	1.898e-51	-0.126	0.558	-0.07	[-0.115; -0.025]	918
HIS	Dynameomics (BBIND)	7.766e-34	-0.015	0.557	-0.009	[-0.053; 0.035]	918
ILE	Dunbrack (BBDEP)	0.002381	0.177	0.195	0.035	[0.019; 0.05]	906
ILE	Dynameomics (BBDEP)	2.056e-24	-0.013	0.196	-0.003	[-0.019; 0.014]	906
ILE	Ultimate (BBIND)	1.511e-11	0.052	0.199	0.01	[-0.006; 0.026]	912
ILE	Dynameomics (BBIND)	6.94e-26	-0.055	0.198	-0.011	[-0.027; 0.006]	912
LEU	Dunbrack (BBDEP)	2.514e-81	-0.754	0.13	-0.098	[-0.108; -0.087]	952
LEU	Dynameomics (BBDEP)	5.459e-121	-1.046 *	0.137	-0.143 **	[-0.155; -0.132]	952
LEU	Ultimate (BBIND)	4.153e-118	-0.969 *	0.133	-0.128 **	[-0.139; -0.118]	953
LEU	Dynameomics (BBIND)	1.935e-130	-1.117 *	0.147	-0.164 **	[-0.176; -0.152]	953
LYS	Dunbrack (BBDEP)	5.819e-26	-0.342	0.237	-0.081	[-0.1; -0.062]	879
LYS	Dynameomics (BBDEP)	1.372e-40	-0.449	0.24	-0.108	[-0.128; -0.088]	879
LYS	Ultimate (BBIND)	3.982e-34	-0.392	0.244	-0.096	[-0.115; -0.076]	891
LYS	Dynameomics (BBIND)	3.548e-55	-0.497	0.245	-0.122	[-0.141; -0.102]	891
MET	Dunbrack (BBDEP)	1.951e-70	-0.915 *	0.203	-0.185 **	[-0.203; -0.168]	704
MET	Dynameomics (BBDEP)	8.59e-95	-1.148 *	0.211	-0.242 **	[-0.26; -0.225]	704
MET	Ultimate (BBIND)	1.627e-80	-0.983 *	0.207	-0.203 **	[-0.221; -0.186]	742
MET	Dynameomics (BBIND)	1.057e-98	-1.139 *	0.21	-0.239 **	[-0.256; -0.222]	742
PHE	Dunbrack (BBDEP)	7.606e-38	-0.371	0.292	-0.108	[-0.132; -0.084]	945
PHE	Dynameomics (BBDEP)	1.074e-86	-0.666 *	0.304	-0.202 **	[-0.228; -0.177]	945
PHE	Ultimate (BBIND)	3.332e-86	-0.666 *	0.308	-0.205 **	[-0.23; -0.18]	952
PHE	Dynameomics (BBIND)	3.507e-99	-0.757 *	0.304	-0.23 **	[-0.255; -0.205]	952
SER	Dunbrack (BBDEP)	1.386e-12	0.195	0.403	0.078	[0.044; 0.113]	882

SER	Dynameomics (BBDEP)	1.782e-73	-0.419	0.43	-0.18	[-0.214; -0.145]	882
SER	Ultimate (BBIND)	2.678e-15	0.178	0.402	0.071	[0.037; 0.105]	893
SER	Dynameomics (BBIND)	3.337e-78	-0.473	0.424	-0.201	[-0.235; -0.167]	893
TRP	Dunbrack (BBDEP)	2.601e-26	-0.129	0.546	-0.071	[-0.118; -0.023]	854
TRP	Dynameomics (BBDEP)	6.507e-69	-0.449	0.571	-0.256	[-0.304; -0.208]	854
TRP	Ultimate (BBIND)	2.167e-77	-0.518 *	0.582	-0.301 **	[-0.35; -0.252]	857
TRP	Dynameomics (BBIND)	7.575e-74	-0.476	0.571	-0.272	[-0.319; -0.225]	857
THR	Dunbrack (BBDEP)	2.514e-4	-0.165	0.124	-0.021	[-0.03; -0.012]	930
THR	Dynameomics (BBDEP)	5.986e-147	-1.805 *	0.194	-0.351 **	[-0.365; -0.336]	930
THR	Ultimate (BBIND)	2.558e-10	-0.219	0.127	-0.028	[-0.037; -0.019]	940
THR	Dynameomics (BBIND)	1.277e-152	-2.139 *	0.185	-0.396 **	[-0.409; -0.383]	940
TYR	Dunbrack (BBDEP)	2.682e-47	-0.26	0.497	-0.129	[-0.17; -0.089]	941
TYR	Dynameomics (BBDEP)	2.664e-81	-0.396	0.5	-0.198	[-0.237; -0.159]	941
TYR	Ultimate (BBIND)	2.591e-77	-0.402	0.499	-0.201	[-0.241; -0.16]	945
TYR	Dynameomics (BBIND)	3.231e-89	-0.455	0.5	-0.227	[-0.267; -0.188]	945
VAL	Dunbrack (BBDEP)	1.238e-12	0.229	0.114	0.026	[0.018; 0.034]	937
VAL	Dynameomics (BBDEP)	8.293e-10	-0.228	0.136	-0.031	[-0.04; -0.022]	937
VAL	Ultimate (BBIND)	0.01569	0.071	0.117	0.008	[0.001; 0.016]	941
VAL	Dynameomics (BBIND)	6.299e-8	-0.286	0.132	-0.038	[-0.044; -0.032]	941

7.6 Data set 19153

Table S7: The results of paired statistics for bcRMSD comparisons between *rotag* and other libraries. * - Cohen's D ≥ 0.5 SD, ** - |Δmean| ≥ 0.1 Å, are considered to be significant.

Residue	Rotamer library	Wilcoxon's p-value	Cohen's D, SD	SD, Å	Δmean, Å	95% CI, Å	N
ARG	Dunbrack (BBDEP)	3.967e-8	0.489	0.744	0.364	[0.299; 0.428]	906
ARG	Dynameomics (BBDEP)	0.001682	0.436	0.745	0.325	[0.261; 0.389]	906
ARG	Ultimate (BBIND)	4.415e-6	0.469	0.745	0.349	[0.285; 0.413]	914
ARG	Dynameomics (BBIND)	0.01495	0.425	0.742	0.316	[0.251; 0.38]	914
ASP	Dunbrack (BBDEP)	1.478e-44	0.561 *	0.477	0.268 **	[0.226; 0.309]	898
ASP	Dynameomics (BBDEP)	0.3966	0.292	0.461	0.135	[0.091; 0.178]	898
ASP	Ultimate (BBIND)	0.001694	0.309	0.498	0.154	[0.112; 0.196]	939
ASP	Dynameomics (BBIND)	0.2883	0.296	0.482	0.142	[0.1; 0.185]	939
ASN	Dunbrack (BBDEP)	1.34e-8	0.348	0.44	0.153	[0.117; 0.189]	934
ASN	Dynameomics (BBDEP)	0.9111	0.226	0.437	0.099	[0.063; 0.134]	934
ASN	Ultimate (BBIND)	4.612e-30	-0.23	0.461	-0.106	[-0.143; -0.069]	941
ASN	Dynameomics (BBIND)	0.003213	0.143	0.434	0.062	[0.026; 0.098]	941
CYS	Dunbrack (BBDEP)	1.206e-10	0.187	0.508	0.095	[0.051; 0.139]	718
CYS	Dynameomics (BBDEP)	1.455e-22	0.111	0.509	0.056	[0.012; 0.101]	718
CYS	Ultimate (BBIND)	2.517e-19	0.142	0.491	0.07	[0.027; 0.112]	768
CYS	Dynameomics (BBIND)	1.077e-35	0.064	0.494	0.032	[-0.011; 0.074]	768
GLU	Dunbrack (BBDEP)	0.9016	0.244	0.418	0.102	[0.066; 0.138]	887
GLU	Dynameomics (BBDEP)	4.682e-13	0.059	0.421	0.025	[-0.011; 0.06]	887
GLU	Ultimate (BBIND)	6.744e-16	-0.049	0.419	-0.021	[-0.059; 0.018]	904
GLU	Dynameomics (BBIND)	1.223e-16	0	0.428	0	[-0.035; 0.034]	904
GLN	Dunbrack (BBDEP)	9.223e-18	-0.119	0.342	-0.041	[-0.067; -0.014]	914
GLN	Dynameomics (BBDEP)	5.418e-53	-0.295	0.336	-0.099	[-0.126; -0.073]	914
GLN	Ultimate (BBIND)	4.276e-75	-0.689 *	0.375	-0.258 **	[-0.29; -0.226]	921
GLN	Dynameomics (BBIND)	9.085e-57	-0.307	0.353	-0.109	[-0.135; -0.082]	921
HIS	Dunbrack (BBDEP)	5.195e-20	-0.03	0.458	-0.014	[-0.053; 0.026]	837
HIS	Dynameomics (BBDEP)	1.981e-25	-0.058	0.458	-0.027	[-0.066; 0.012]	837
HIS	Ultimate (BBIND)	2.115e-53	-0.232	0.462	-0.107	[-0.145; -0.069]	906
HIS	Dynameomics (BBIND)	3.151e-38	-0.11	0.462	-0.051	[-0.087; -0.014]	906
ILE	Dunbrack (BBDEP)	2.309e-6	0.134	0.194	0.026	[0.01; 0.042]	914
ILE	Dynameomics (BBDEP)	1.565e-29	-0.041	0.195	-0.008	[-0.024; 0.008]	914
ILE	Ultimate (BBIND)	1.445e-13	0.024	0.199	0.005	[-0.011; 0.021]	921
ILE	Dynameomics (BBIND)	7.287e-32	-0.098	0.199	-0.019	[-0.036; -0.003]	921
LEU	Dunbrack (BBDEP)	6.642e-85	-0.579 *	0.175	-0.101 **	[-0.112; -0.09]	951
LEU	Dynameomics (BBDEP)	3.477e-118	-0.826 *	0.175	-0.144 **	[-0.156; -0.133]	951
LEU	Ultimate (BBIND)	2.882e-116	-0.729 *	0.18	-0.132 **	[-0.143; -0.12]	953
LEU	Dynameomics (BBIND)	1.9e-126	-0.9 *	0.18	-0.162 **	[-0.174; -0.15]	953
LYS	Dunbrack (BBDEP)	1.071e-26	-0.344	0.237	-0.082	[-0.101; -0.062]	882
LYS	Dynameomics (BBDEP)	5.611e-43	-0.47	0.24	-0.113	[-0.132; -0.093]	882
LYS	Ultimate (BBIND)	2.551e-31	-0.367	0.243	-0.089	[-0.109; -0.069]	892
LYS	Dynameomics (BBIND)	3.373e-50	-0.482	0.247	-0.119	[-0.139; -0.099]	892
MET	Dunbrack (BBDEP)	1.023e-65	-0.894 *	0.196	-0.176 **	[-0.193; -0.158]	702
MET	Dynameomics (BBDEP)	1.353e-94	-1.123 *	0.218	-0.245 **	[-0.263; -0.226]	702
MET	Ultimate (BBIND)	1.414e-79	-0.926 *	0.214	-0.199 **	[-0.218; -0.179]	734
MET	Dynameomics (BBIND)	5.739e-99	-1.111 *	0.224	-0.249 **	[-0.27; -0.229]	734
PHE	Dunbrack (BBDEP)	8.527e-42	-0.349	0.317	-0.11	[-0.135; -0.086]	952
PHE	Dynameomics (BBDEP)	2.843e-95	-0.658 *	0.318	-0.209 **	[-0.235; -0.184]	952
PHE	Ultimate (BBIND)	1.048e-88	-0.609 *	0.324	-0.197 **	[-0.223; -0.172]	959
PHE	Dynameomics (BBIND)	1.028e-106	-0.717 *	0.32	-0.229 **	[-0.255; -0.204]	959
SER	Dunbrack (BBDEP)	2.981e-12	0.235	0.439	0.103	[0.067; 0.14]	901

SER	Dynameomics (BBDEP)	1.625e-62	-0.282	0.456	-0.129	[-0.166; -0.091]	901
SER	Ultimate (BBIND)	2.32e-14	0.217	0.442	0.096	[0.059; 0.132]	904
SER	Dynameomics (BBIND)	5.567e-67	-0.35	0.458	-0.161	[-0.197; -0.124]	904
TRP	Dunbrack (BBDEP)	2.174e-26	-0.133	0.504	-0.067	[-0.113; -0.021]	859
TRP	Dynameomics (BBDEP)	1.096e-60	-0.446	0.533	-0.238	[-0.286; -0.189]	859
TRP	Ultimate (BBIND)	2.715e-74	-0.543 *	0.545	-0.296 **	[-0.344; -0.247]	862
TRP	Dynameomics (BBIND)	3.74e-70	-0.507 *	0.534	-0.271 **	[-0.319; -0.222]	862
THR	Dunbrack (BBDEP)	4.975e-6	-0.206	0.112	-0.023	[-0.031; -0.015]	927
THR	Dynameomics (BBDEP)	5.258e-147	-1.877 *	0.183	-0.344 **	[-0.358; -0.331]	927
THR	Ultimate (BBIND)	8.061e-15	-0.258	0.114	-0.03	[-0.037; -0.022]	940
THR	Dynameomics (BBIND)	1.283e-152	-2.163 *	0.177	-0.383 **	[-0.395; -0.37]	940
TYR	Dunbrack (BBDEP)	7.249e-45	-0.23	0.491	-0.113	[-0.156; -0.071]	943
TYR	Dynameomics (BBDEP)	4.715e-82	-0.385	0.493	-0.19	[-0.233; -0.147]	943
TYR	Ultimate (BBIND)	3.52e-81	-0.408	0.498	-0.203	[-0.245; -0.16]	950
TYR	Dynameomics (BBIND)	3.278e-94	-0.466	0.494	-0.23	[-0.273; -0.187]	950
VAL	Dunbrack (BBDEP)	4.434e-13	0.22	0.112	0.025	[0.017; 0.033]	935
VAL	Dynameomics (BBDEP)	3.819e-17	-0.321	0.133	-0.043	[-0.052; -0.033]	936
VAL	Ultimate (BBIND)	0.09803	0.039	0.118	0.005	[-0.004; 0.013]	939
VAL	Dynameomics (BBIND)	4.093e-15	-0.358	0.133	-0.048	[-0.055; -0.041]	939

7.7 Data set 25759

Table S8: The results of paired statistics for bcRMSD comparisons between *rotag* and other libraries. * - Cohen's D ≥ 0.5 SD, ** - |Δmean| ≥ 0.1 Å, are considered to be significant.

Residue	Rotamer library	Wilcoxon's p-value	Cohen's D, SD	SD, Å	Δmean, Å	95% CI, Å	N
ARG	Dunbrack (BBDEP)	4.958e-10	0.525 *	0.784	0.412 **	[0.344; 0.48]	909
ARG	Dynameomics (BBDEP)	2.351e-4	0.477	0.778	0.371	[0.302; 0.439]	909
ARG	Ultimate (BBIND)	1.253e-8	0.493	0.794	0.392	[0.325; 0.459]	916
ARG	Dynameomics (BBIND)	3.932e-4	0.465	0.78	0.363	[0.295; 0.431]	916
ASP	Dunbrack (BBDEP)	1.809e-51	0.597 *	0.421	0.251 **	[0.215; 0.287]	928
ASP	Dynameomics (BBDEP)	0.005477	0.311	0.406	0.126	[0.088; 0.165]	929
ASP	Ultimate (BBIND)	2.136e-4	0.287	0.43	0.124	[0.087; 0.16]	948
ASP	Dynameomics (BBIND)	0.03296	0.291	0.416	0.121	[0.084; 0.158]	948
ASN	Dunbrack (BBDEP)	6.656e-20	0.429	0.396	0.17	[0.137; 0.204]	929
ASN	Dynameomics (BBDEP)	0.09472	0.26	0.394	0.102	[0.069; 0.136]	929
ASN	Ultimate (BBIND)	1.27e-17	-0.174	0.427	-0.074	[-0.108; -0.04]	943
ASN	Dynameomics (BBIND)	0.8593	0.193	0.399	0.077	[0.044; 0.11]	943
CYS	Dunbrack (BBDEP)	2.425e-16	0.122	0.48	0.059	[0.018; 0.099]	751
CYS	Dynameomics (BBDEP)	4.188e-35	0.046	0.472	0.022	[-0.019; 0.063]	751
CYS	Ultimate (BBIND)	1.268e-27	0.079	0.472	0.037	[-0.002; 0.077]	781
CYS	Dynameomics (BBIND)	2.537e-40	0.009	0.47	0.004	[-0.036; 0.044]	781
GLU	Dunbrack (BBDEP)	0.9844	0.253	0.429	0.108	[0.072; 0.144]	892
GLU	Dynameomics (BBDEP)	2.191e-10	0.081	0.429	0.035	[-0.001; 0.07]	892
GLU	Ultimate (BBIND)	9.84e-14	-0.035	0.431	-0.015	[-0.054; 0.023]	912
GLU	Dynameomics (BBIND)	1.683e-15	0.035	0.434	0.015	[-0.019; 0.05]	912
GLN	Dunbrack (BBDEP)	1.966e-15	-0.038	0.362	-0.014	[-0.044; 0.016]	917
GLN	Dynameomics (BBDEP)	3.047e-46	-0.22	0.358	-0.079	[-0.108; -0.049]	917
GLN	Ultimate (BBIND)	8.159e-73	-0.641 *	0.388	-0.248 **	[-0.282; -0.214]	925
GLN	Dynameomics (BBIND)	1.64e-51	-0.256	0.369	-0.094	[-0.124; -0.065]	924
HIS	Dunbrack (BBDEP)	2.256e-22	-0.017	0.544	-0.009	[-0.051; 0.032]	847
HIS	Dynameomics (BBDEP)	4.14e-32	-0.04	0.535	-0.021	[-0.064; 0.021]	847
HIS	Ultimate (BBIND)	4.514e-51	-0.156	0.556	-0.087	[-0.129; -0.044]	916
HIS	Dynameomics (BBIND)	1.728e-38	-0.052	0.547	-0.028	[-0.071; 0.014]	916
ILE	Dunbrack (BBDEP)	0.002128	0.2	0.19	0.038	[0.022; 0.054]	913
ILE	Dynameomics (BBDEP)	2.098e-24	0.011	0.189	0.002	[-0.015; 0.019]	913
ILE	Ultimate (BBIND)	3.461e-14	0.038	0.192	0.007	[-0.009; 0.024]	920
ILE	Dynameomics (BBIND)	6.746e-29	-0.057	0.191	-0.011	[-0.028; 0.006]	920
LEU	Dunbrack (BBDEP)	2.979e-76	-0.577	0.162	-0.093	[-0.105; -0.082]	945
LEU	Dynameomics (BBDEP)	3.39e-117	-0.821 *	0.174	-0.143 **	[-0.154; -0.131]	945
LEU	Ultimate (BBIND)	1.5e-110	-0.757 *	0.167	-0.126 **	[-0.137; -0.115]	948
LEU	Dynameomics (BBIND)	4.56e-125	-0.898 *	0.179	-0.161 **	[-0.173; -0.149]	948
LYS	Dunbrack (BBDEP)	7.125e-20	-0.262	0.23	-0.06	[-0.08; -0.041]	894
LYS	Dynameomics (BBDEP)	2.249e-31	-0.364	0.234	-0.085	[-0.105; -0.066]	894
LYS	Ultimate (BBIND)	1.269e-24	-0.304	0.237	-0.072	[-0.092; -0.053]	903
LYS	Dynameomics (BBIND)	3.661e-40	-0.401	0.241	-0.097	[-0.116; -0.078]	903
MET	Dunbrack (BBDEP)	1.233e-71	-0.966 *	0.191	-0.185 **	[-0.202; -0.167]	707
MET	Dynameomics (BBDEP)	7.33e-99	-1.227 *	0.203	-0.249 **	[-0.266; -0.232]	707
MET	Ultimate (BBIND)	6.497e-81	-0.956 *	0.209	-0.2 **	[-0.22; -0.18]	745
MET	Dynameomics (BBIND)	5.535e-99	-1.173 *	0.214	-0.251 **	[-0.272; -0.231]	745
PHE	Dunbrack (BBDEP)	9.902e-43	-0.306	0.336	-0.103	[-0.132; -0.074]	943
PHE	Dynameomics (BBDEP)	3.32e-87	-0.554 *	0.345	-0.191 **	[-0.22; -0.163]	943
PHE	Ultimate (BBIND)	2.956e-87	-0.563 *	0.347	-0.195 **	[-0.224; -0.166]	949
PHE	Dynameomics (BBIND)	2.428e-95	-0.627 *	0.346	-0.217 **	[-0.246; -0.188]	949
SER	Dunbrack (BBDEP)	2.273e-16	0.213	0.426	0.091	[0.055; 0.126]	899

SER	Dynameomics (BBDEP)	8.857e-70	-0.374	0.447	-0.167	[-0.203; -0.132]	899
SER	Ultimate (BBIND)	4.849e-20	0.191	0.419	0.08	[0.045; 0.115]	915
SER	Dynameomics (BBIND)	2.176e-73	-0.437	0.441	-0.193	[-0.228; -0.158]	915
TRP	Dunbrack (BBDEP)	1.828e-30	-0.105	0.581	-0.061	[-0.114; -0.008]	862
TRP	Dynameomics (BBDEP)	2.013e-66	-0.379	0.614	-0.232	[-0.285; -0.18]	862
TRP	Ultimate (BBIND)	4.718e-82	-0.496	0.621	-0.308	[-0.363; -0.252]	868
TRP	Dynameomics (BBIND)	8.552e-74	-0.427	0.614	-0.262	[-0.315; -0.21]	868
THR	Dunbrack (BBDEP)	3.1e-4	-0.137	0.125	-0.017	[-0.025; -0.009]	934
THR	Dynameomics (BBDEP)	3.503e-146	-1.781 *	0.193	-0.343 **	[-0.357; -0.329]	934
THR	Ultimate (BBIND)	2.286e-10	-0.196	0.126	-0.025	[-0.033; -0.017]	942
THR	Dynameomics (BBIND)	1.386e-151	-2.118 *	0.182	-0.385 **	[-0.398; -0.372]	942
TYR	Dunbrack (BBDEP)	6.504e-59	-0.31	0.489	-0.152	[-0.194; -0.109]	936
TYR	Dynameomics (BBDEP)	1.137e-90	-0.45	0.491	-0.221	[-0.263; -0.18]	936
TYR	Ultimate (BBIND)	7.55e-92	-0.473	0.497	-0.235	[-0.277; -0.193]	942
TYR	Dynameomics (BBIND)	1.021e-103	-0.521 *	0.499	-0.26 **	[-0.301; -0.218]	942
VAL	Dunbrack (BBDEP)	8.408e-14	0.26	0.113	0.029	[0.021; 0.037]	956
VAL	Dynameomics (BBDEP)	1.195e-14	-0.288	0.142	-0.041	[-0.051; -0.031]	956
VAL	Ultimate (BBIND)	0.02844	0.091	0.115	0.01	[0.002; 0.019]	958
VAL	Dynameomics (BBIND)	2.07e-13	-0.326	0.13	-0.042	[-0.049; -0.035]	958

7.8 Data set 29821

Table S9: The results of paired statistics for bcRMSD comparisons between *rotag* and other libraries. * - Cohen's D $\geq$ 0.5 SD, ** - $|\Delta$mean$|$ $\geq$ 0.1 Å, are considered to be significant.

Residue	Rotamer library	Wilcoxon's p-value	Cohen's D, SD	SD, Å	Δmean, Å	95% CI, Å	N
ARG	Dunbrack (BBDEP)	3.6e-15	0.515 *	0.751	0.387 **	[0.325; 0.45]	914[illegible]
ARG	Dynameomics (BBDEP)	2.566e-6	0.454	0.75	0.341	[0.278; 0.404]	914[illegible]
ARG	Ultimate (BBIND)	2.812e-11	0.488	0.755	0.368	[0.306; 0.43]	918[illegible]
ARG	Dynameomics (BBIND)	7.969e-6	0.451	0.75	0.338	[0.276; 0.401]	918[illegible]
ASP	Dunbrack (BBDEP)	4.148e-45	0.568 *	0.411	0.234 **	[0.198; 0.27]	913[illegible]
ASP	Dynameomics (BBDEP)	0.1469	0.259	0.406	0.105	[0.067; 0.143]	914[illegible]
ASP	Ultimate (BBIND)	0.04295	0.239	0.419	0.1	[0.064; 0.136]	938[illegible]
ASP	Dynameomics (BBIND)	0.1543	0.26	0.408	0.106	[0.07; 0.143]	938[illegible]
ASN	Dunbrack (BBDEP)	9.084e-11	0.373	0.408	0.152	[0.118; 0.187]	923[illegible]
ASN	Dynameomics (BBDEP)	0.908	0.215	0.408	0.088	[0.054; 0.122]	923[illegible]
ASN	Ultimate (BBIND)	6.842e-26	-0.236	0.441	-0.104	[-0.139; -0.068]	941[illegible]
ASN	Dynameomics (BBIND)	0.005444	0.136	0.417	0.057	[0.022; 0.092]	941[illegible]
CYS	Dunbrack (BBDEP)	2.662e-14	0.158	0.477	0.075	[0.033; 0.118]	725[illegible]
CYS	Dynameomics (BBDEP)	1.64e-29	0.078	0.477	0.037	[-0.005; 0.08]	725[illegible]
CYS	Ultimate (BBIND)	1.532e-28	0.102	0.464	0.047	[0.007; 0.088]	762[illegible]
CYS	Dynameomics (BBIND)	7.694e-41	0.02	0.462	0.009	[-0.032; 0.051]	762[illegible]
GLU	Dunbrack (BBDEP)	0.5215	0.248	0.418	0.104	[0.068; 0.139]	898[illegible]
GLU	Dynameomics (BBDEP)	4.117e-18	0.032	0.418	0.013	[-0.022; 0.049]	898[illegible]
GLU	Ultimate (BBIND)	9.186e-18	-0.066	0.423	-0.028	[-0.066; 0.01]	919[illegible]
GLU	Dynameomics (BBIND)	1.572e-20	-0.011	0.429	-0.005	[-0.038; 0.029]	919[illegible]
GLN	Dunbrack (BBDEP)	1.34e-20	-0.084	0.347	-0.029	[-0.054; -0.004]	924[illegible]
GLN	Dynameomics (BBDEP)	1.327e-54	-0.277	0.343	-0.095	[-0.12; -0.07]	925[illegible]
GLN	Ultimate (BBIND)	1.1e-80	-0.696 *	0.371	-0.258 **	[-0.288; -0.227]	931[illegible]
GLN	Dynameomics (BBIND)	3.184e-61	-0.337	0.346	-0.116	[-0.141; -0.092]	931[illegible]
HIS	Dunbrack (BBDEP)	5.021e-24	-0.033	0.479	-0.016	[-0.058; 0.027]	858[illegible]
HIS	Dynameomics (BBDEP)	4.49e-37	-0.077	0.476	-0.037	[-0.078; 0.005]	858[illegible]
HIS	Ultimate (BBIND)	3.734e-55	-0.188	0.499	-0.094	[-0.136; -0.051]	914[illegible]
HIS	Dynameomics (BBIND)	2.61e-44	-0.084	0.499	-0.042	[-0.083; -0.001]	914[illegible]
ILE	Dunbrack (BBDEP)	0.003084	0.183	0.195	0.036	[0.02; 0.051]	930[illegible]
ILE	Dynameomics (BBDEP)	3.307e-21	0.022	0.194	0.004	[-0.012; 0.021]	930[illegible]
ILE	Ultimate (BBIND)	5.054e-12	0.066	0.201	0.013	[-0.002; 0.029]	931[illegible]
ILE	Dynameomics (BBIND)	7.649e-26	-0.038	0.198	-0.008	[-0.024; 0.009]	931[illegible]
LEU	Dunbrack (BBDEP)	4.824e-73	-0.393	0.215	-0.085	[-0.1; -0.069]	951[illegible]
LEU	Dynameomics (BBDEP)	1.392e-114	-0.613 *	0.219	-0.134 **	[-0.15; -0.118]	951[illegible]
LEU	Ultimate (BBIND)	2.38e-101	-0.498	0.226	-0.112	[-0.129; -0.096]	954[illegible]
LEU	Dynameomics (BBIND)	2.053e-121	-0.658 *	0.23	-0.151 **	[-0.169; -0.134]	954[illegible]
LYS	Dunbrack (BBDEP)	9.864e-22	-0.248	0.26	-0.064	[-0.086; -0.043]	898[illegible]
LYS	Dynameomics (BBDEP)	9.338e-34	-0.352	0.263	-0.093	[-0.114; -0.071]	898[illegible]
LYS	Ultimate (BBIND)	1.146e-27	-0.314	0.268	-0.084	[-0.106; -0.063]	908[illegible]
LYS	Dynameomics (BBIND)	6.864e-49	-0.416	0.264	-0.11	[-0.13; -0.089]	908[illegible]
MET	Dunbrack (BBDEP)	7.579e-80	-0.965 *	0.206	-0.199 **	[-0.216; -0.182]	715[illegible]
MET	Dynameomics (BBDEP)	1.728e-94	-1.156 *	0.216	-0.249 **	[-0.267; -0.232]	715[illegible]
MET	Ultimate (BBIND)	3.877e-87	-1.037 *	0.211	-0.219 **	[-0.238; -0.201]	739[illegible]
MET	Dynameomics (BBIND)	8.917e-102	-1.199 *	0.217	-0.26 **	[-0.279; -0.242]	739[illegible]
PHE	Dunbrack (BBDEP)	1.809e-36	-0.246	0.392	-0.096	[-0.129; -0.064]	948[illegible]
PHE	Dynameomics (BBDEP)	3.479e-79	-0.445	0.4	-0.178	[-0.21; -0.145]	948[illegible]
PHE	Ultimate (BBIND)	5.192e-77	-0.441	0.403	-0.178	[-0.21; -0.145]	952[illegible]
PHE	Dynameomics (BBIND)	5.793e-92	-0.524 *	0.4	-0.21 **	[-0.242; -0.177]	952[illegible]
SER	Dunbrack (BBDEP)	1.956e-13	0.209	0.432	0.09	[0.055; 0.126]	888[illegible]

SER	Dynameomics (BBDEP)	4.112e-66	-0.352	0.456	-0.161	[-0.195; -0.126]	888
SER	Ultimate (BBIND)	1.33e-16	0.191	0.427	0.082	[0.047; 0.117]	901
SER	Dynameomics (BBIND)	1.785e-69	-0.403	0.454	-0.183	[-0.217; -0.149]	901
TRP	Dunbrack (BBDEP)	2.1590000000000001e-32	-0.178	0.502	-0.089	[-0.134; -0.044]	875
TRP	Dynameomics (BBDEP)	2.998e-73	-0.486	0.531	-0.258	[-0.305; -0.212]	875
TRP	Ultimate (BBIND)	4.376e-84	-0.589 *	0.541	-0.319 **	[-0.366; -0.272]	881
TRP	Dynameomics (BBIND)	6.134e-79	-0.53 *	0.53	-0.281 **	[-0.328; -0.234]	881
THR	Dunbrack (BBDEP)	6.984e-4	-0.134	0.12	-0.016	[-0.025; -0.007]	934
THR	Dynameomics (BBDEP)	9.585e-145	-1.768 *	0.19	-0.335 **	[-0.35; -0.32]	934
THR	Ultimate (BBIND)	1.351e-7	-0.171	0.122	-0.021	[-0.03; -0.012]	940
THR	Dynameomics (BBIND)	4.9e-151	-2.082 *	0.178	-0.371 **	[-0.384; -0.358]	940
TYR	Dunbrack (BBDEP)	3.389e-44	-0.218	0.495	-0.108	[-0.151; -0.065]	949
TYR	Dynameomics (BBDEP)	1.126e-77	-0.35	0.496	-0.173	[-0.216; -0.13]	949
TYR	Ultimate (BBIND)	4.266e-84	-0.389	0.502	-0.195	[-0.238; -0.153]	953
TYR	Dynameomics (BBIND)	1.11e-91	-0.424	0.5	-0.212	[-0.254; -0.169]	953
VAL	Dunbrack (BBDEP)	2.86e-14	0.262	0.112	0.029	[0.021; 0.037]	933
VAL	Dynameomics (BBDEP)	1.837e-10	-0.267	0.129	-0.034	[-0.044; -0.025]	933
VAL	Ultimate (BBIND)	0.088	0.037	0.121	0.004	[-0.004; 0.013]	940
VAL	Dynameomics (BBIND)	5.426e-14	-0.344	0.135	-0.046	[-0.053; -0.04]	940

8 bcRMSD outliers

8.1 Data set 13097

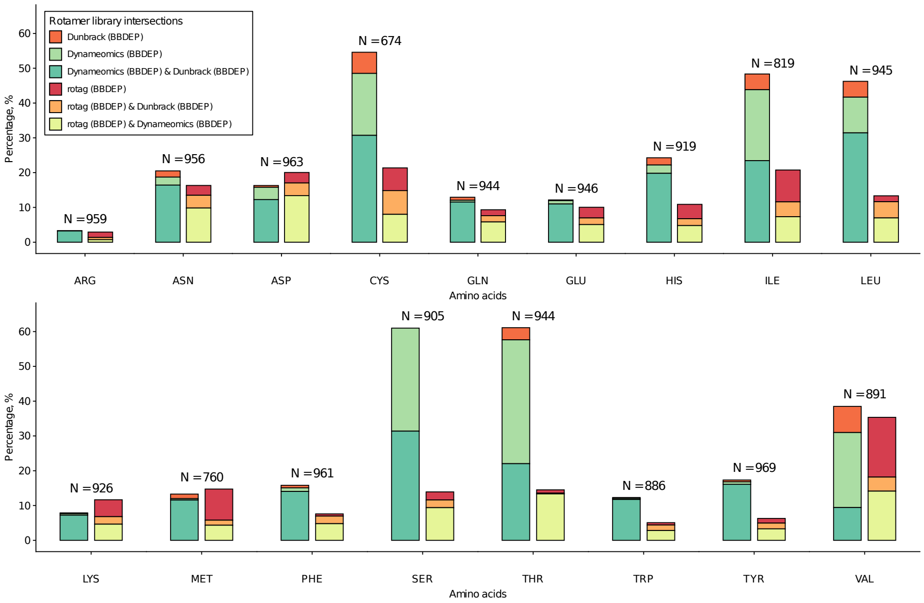

Figure S9: the figure shows stacked barplots that represent outliers of the BB-DEP libraries where only the outliers with bcRMSD values ≥ 0.1 Å are shown. The barplots are divided into two subgroups based on the presence or absence of *rotag* outliers in the groups for better comparison. Any outliers that are common to all three rotamer libraries have been excluded. N depicts the total amount of outliers for each residue in the 13097 dataset.

8.2 Raw data set results

The barplots of the visualised and the rest of the data sets can be generated from raw bcRMSD results stored in Supplementary File *rotamer-library-bc-rmsd.csv* of *SupplementaryData.zip* archive.

9 Best-case dihedral angles

9.1 Data set 13097

9.1.1 ASP

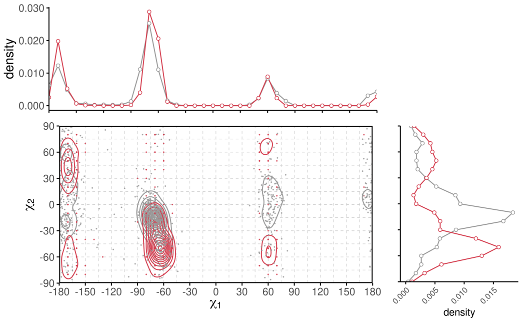

ASP – χ_1 and χ_2 dihedral angles

Figure S10: *rotag* rotamer library

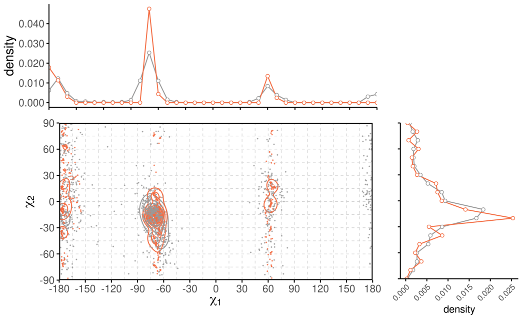

ASP – χ_1 and χ_2 dihedral angles

Figure S11: Dunbrack (BBDEP) rotamer library

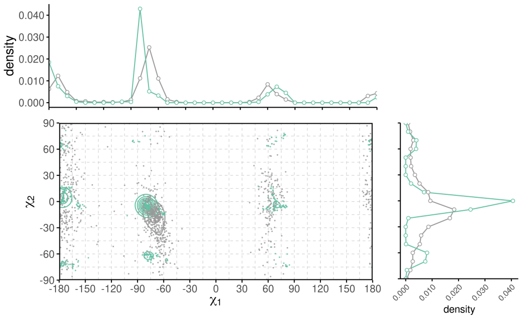

ASP – χ_1 and χ_2 dihedral angles

Figure S12: Dynameomics (BBDEP) rotamer library

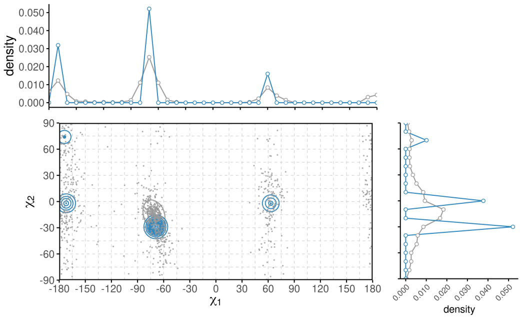

ASP – χ_1 and χ_2 dihedral angles

Figure S13: Ultimate (BBIND) rotamer library

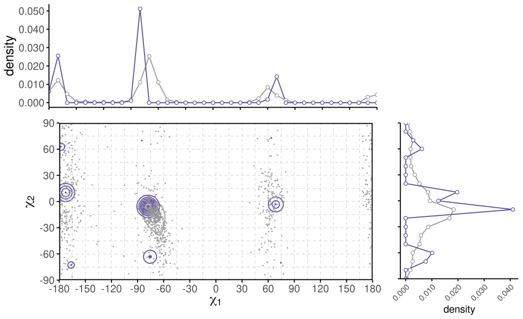

ASP – χ_1 and χ_2 dihedral angles

Figure S14: Dynameomics (BBIND) rotamer library

9.1.2 ASN

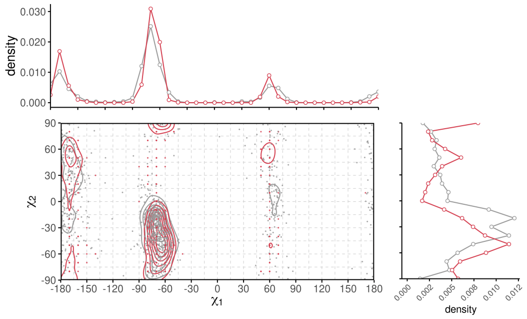

ASN – χ_1 and χ_2 dihedral angles

Figure S15: *rotag* rotamer library

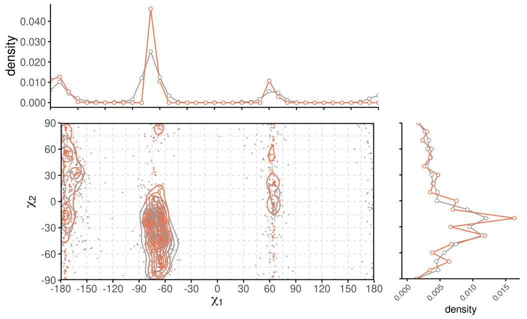

ASN – χ_1 and χ_2 dihedral angles

Figure S16: Dunbrack (BBDEP) rotamer library

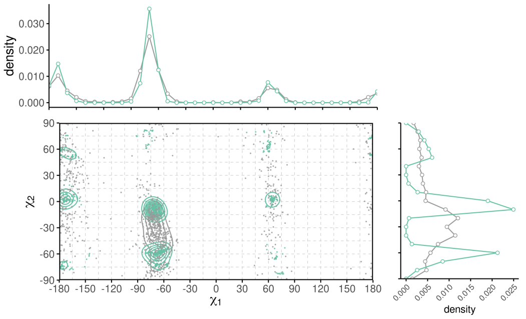

ASN – χ_1 and χ_2 dihedral angles

Figure S17: Dynameomics (BBDEP) rotamer library

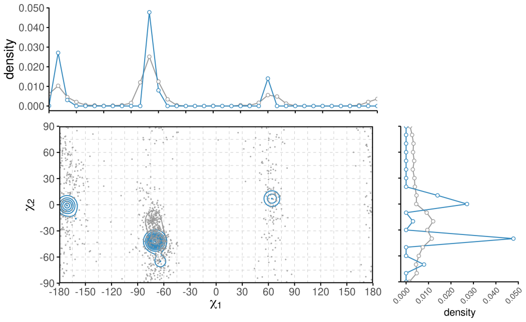

ASN – χ_1 and χ_2 dihedral angles

Figure S18: Ultimate (BBIND) rotamer library

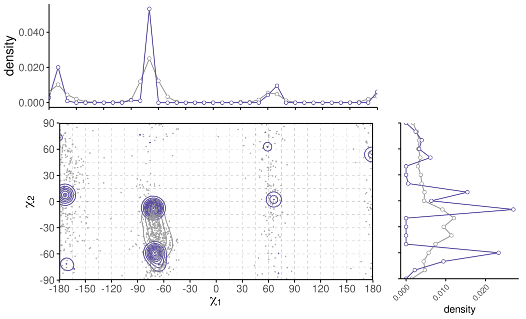

ASN – χ_1 and χ_2 dihedral angles

Figure S19: Dynameomics (BBIND) rotamer library

9.1.3 LEU

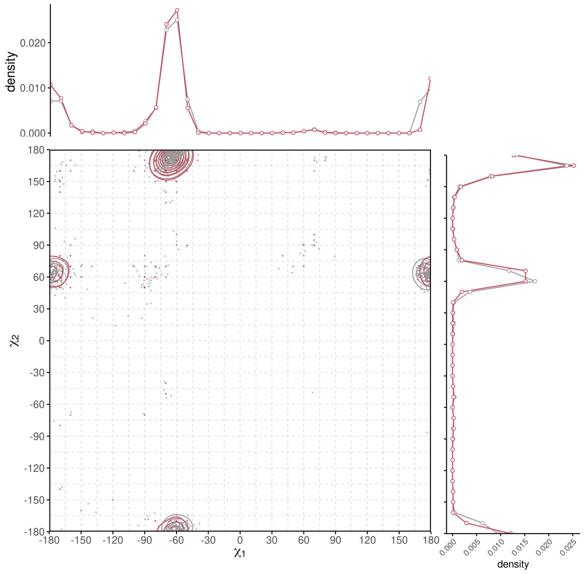

LEU – χ_1 and χ_2 dihedral angles

Figure S20: *rotag* rotamer library

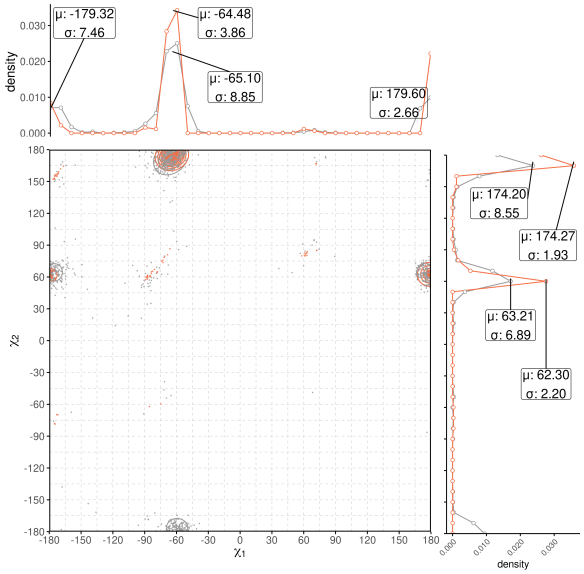

LEU – χ_1 and χ_2 dihedral angles

Figure S21: Dunbrack (BBDEP) rotamer library

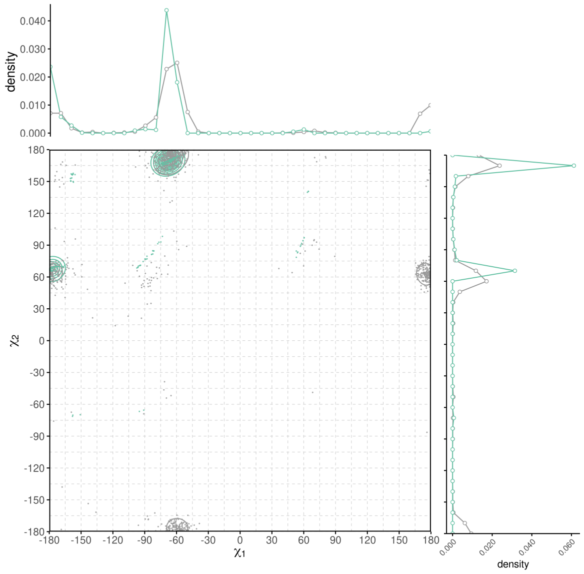

LEU – χ_1 and χ_2 dihedral angles

Figure S22: Dynameomics (BBDEP) rotamer library

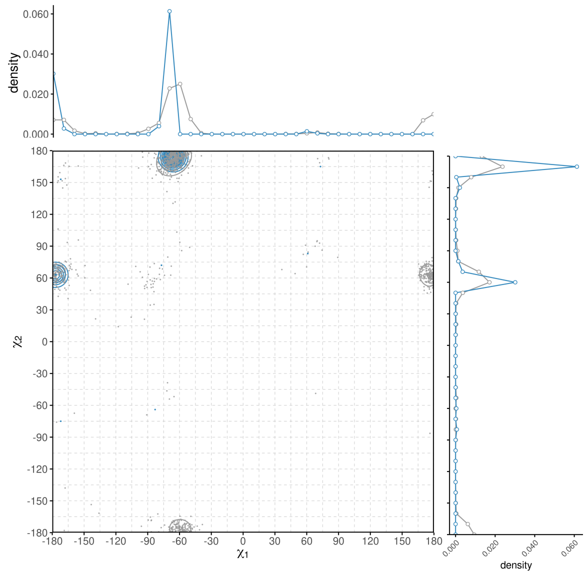

LEU – χ_1 and χ_2 dihedral angles

Figure S23: Ultimate (BBIND) rotamer library

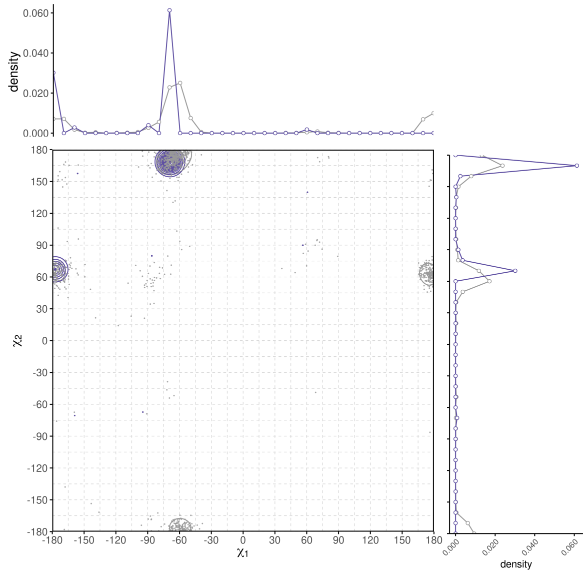

LEU – χ_1 and χ_2 dihedral angles

Figure S24: Dynameomics (BBIND) rotamer library

9.1.4 MET

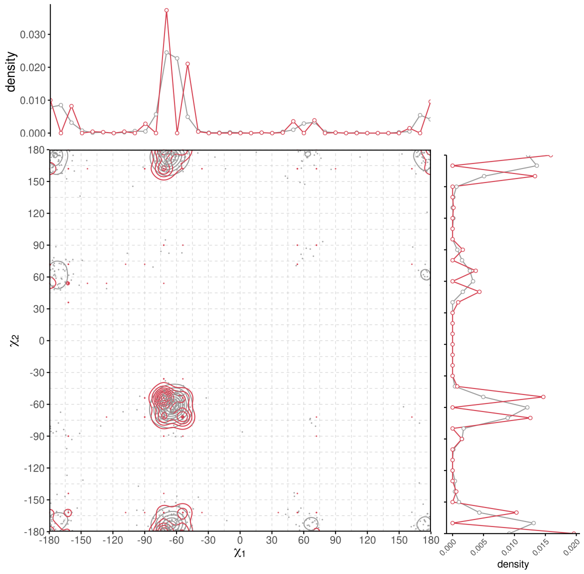

MET – χ_1 and χ_2 dihedral angles

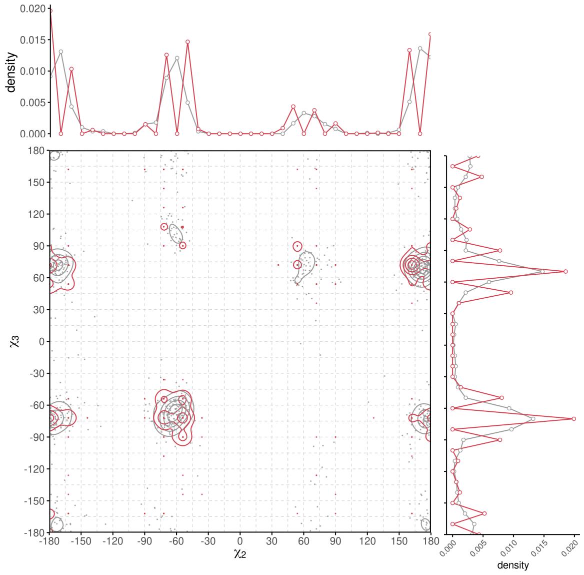

MET – χ_2 and χ_3 dihedral angles

Figure S25: *rotag* rotamer library

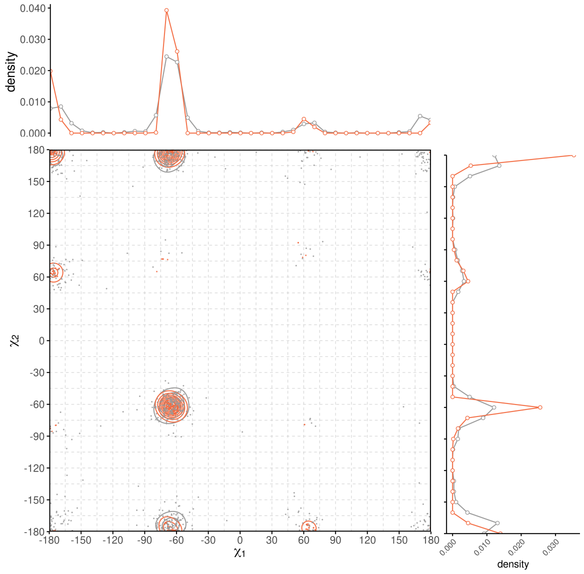

MET – χ_1 and χ_2 dihedral angles

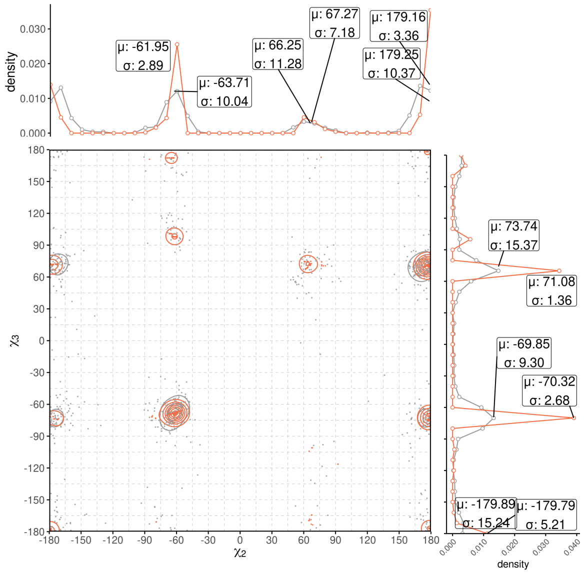

MET – χ_2 and χ_3 dihedral angles

Figure S26: Dunbrack (BBDEP) rotamer library

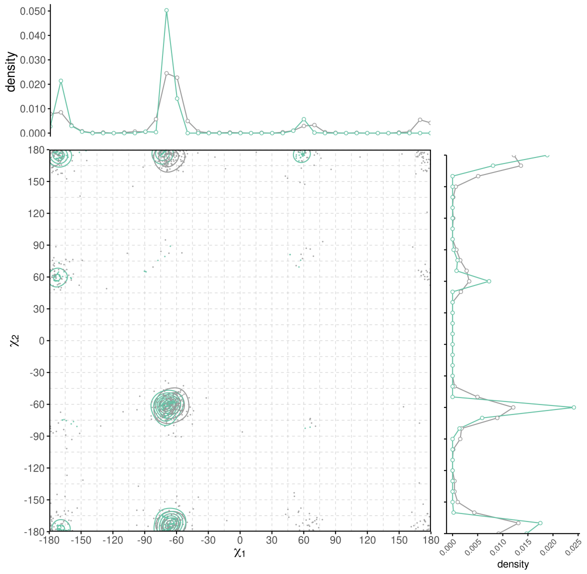

MET – χ_1 and χ_2 dihedral angles

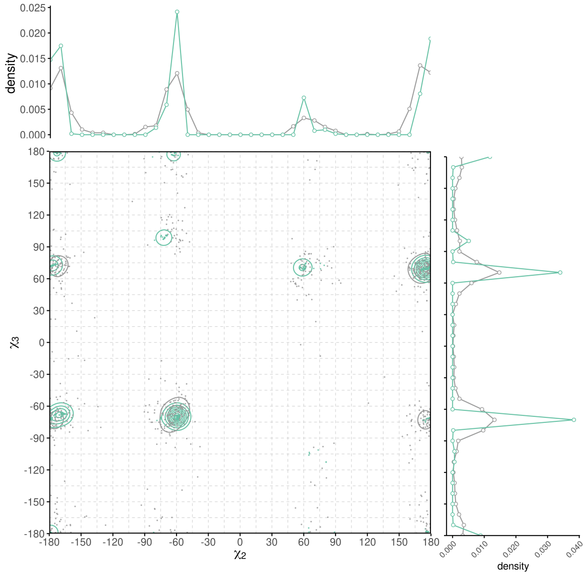

MET – χ_2 and χ_3 dihedral angles

Figure S27: Dynameomics (BBDEP) rotamer library

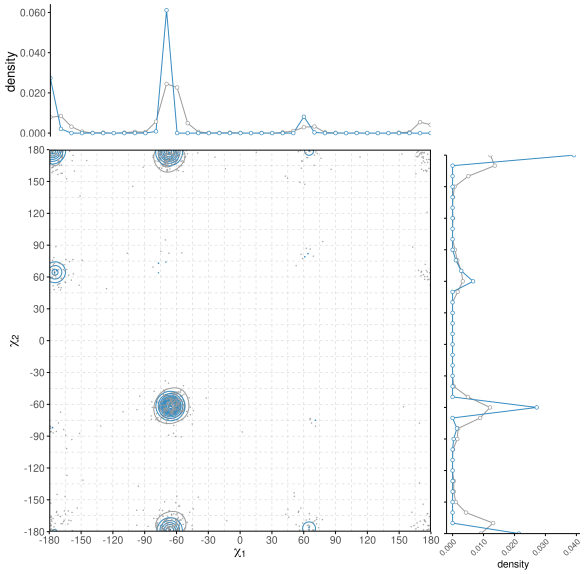

MET – χ_1 and χ_2 dihedral angles

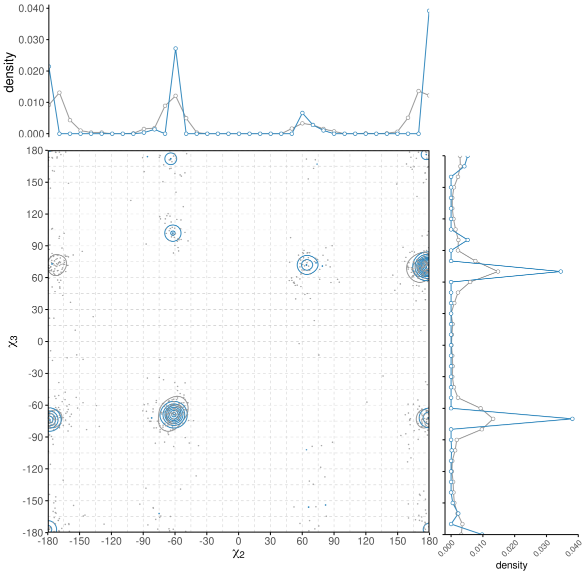

MET – χ_2 and χ_3 dihedral angles

Figure S28: Ultimate (BBIND) rotamer library

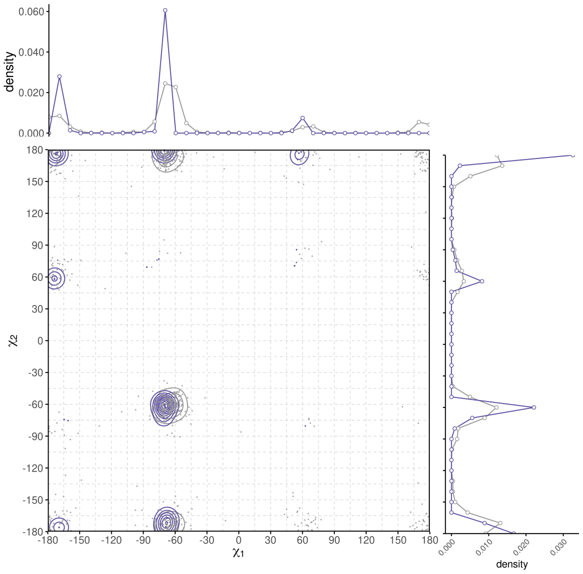

MET – χ_1 and χ_2 dihedral angles

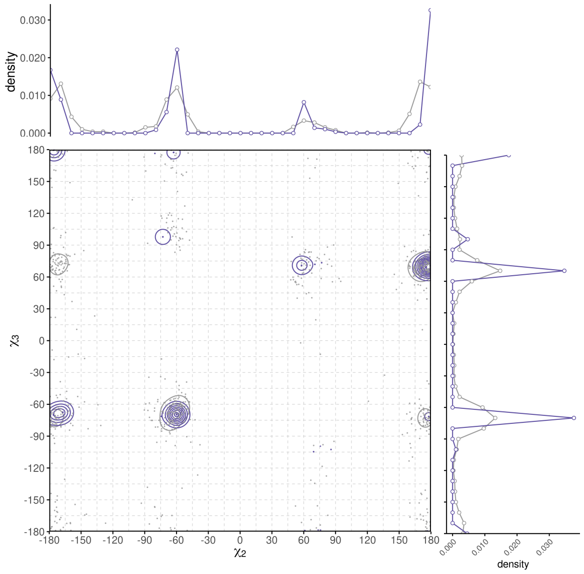

MET – χ_2 and χ_3 dihedral angles

Figure S29: Dynameomics (BBIND) rotamer library

9.1.5 PHE

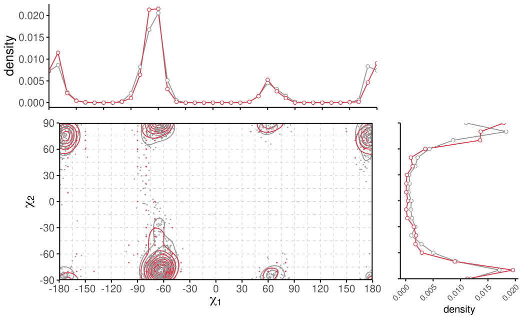

PHE – χ_1 and χ_2 dihedral angles

Figure S30: *rotag* rotamer library

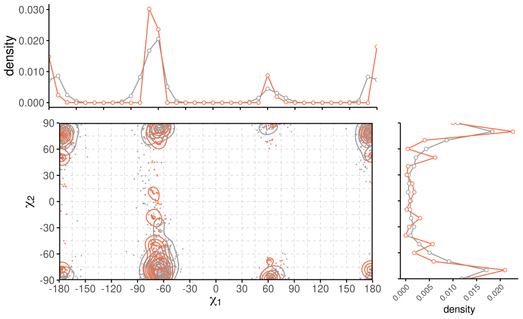

PHE – χ_1 and χ_2 dihedral angles

Figure S31: Dunbrack (BBDEP) rotamer library

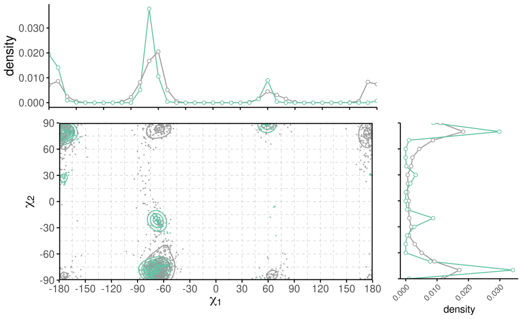

PHE – χ_1 and χ_2 dihedral angles

Figure S32: Dynameomics (BBDEP) rotamer library

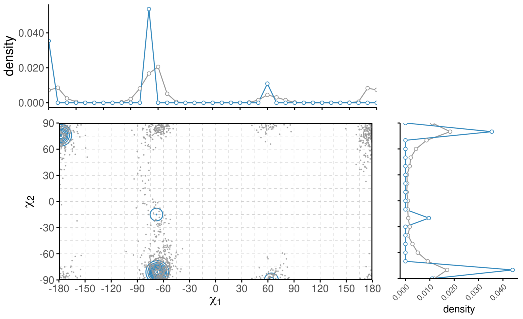

PHE – χ_1 and χ_2 dihedral angles

Figure S33: Ultimate (BBIND) rotamer library

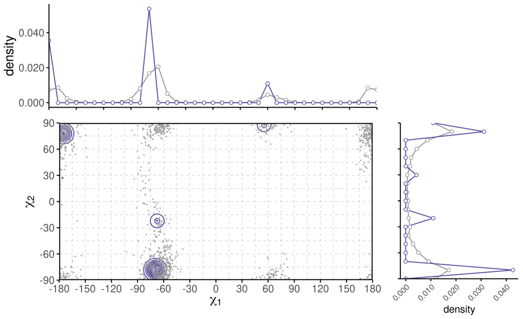

PHE – χ_1 and χ_2 dihedral angles

Figure S34: Dynameomics (BBIND) rotamer library

9.1.6 SER

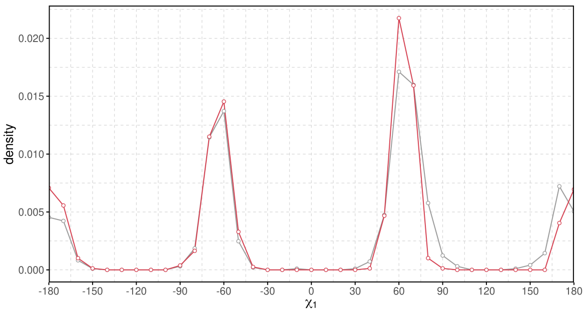

SER – χ_1 dihedral angles

Figure S35: *rotag* rotamer library

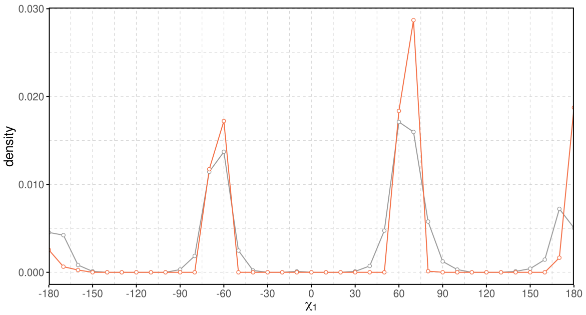

SER – χ_1 dihedral angles

Figure S36: Dunbrack (BBDEP) rotamer library

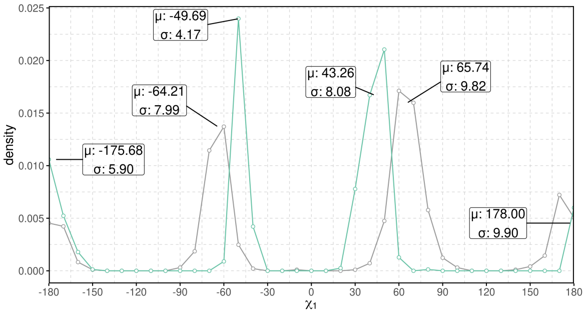

SER – χ_1 dihedral angles

Figure S37: Dynameomics (BBDEP) rotamer library

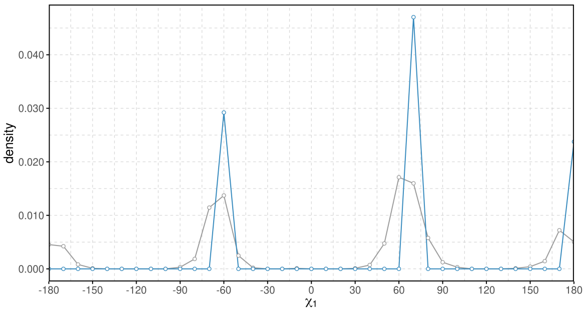

SER – χ_1 dihedral angles

Figure S38: Ultimate (BBIND) rotamer library

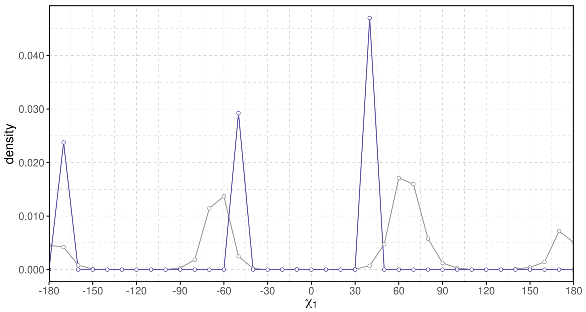

SER – χ_1 dihedral angles

Figure S39: Dynameomics (BBIND) rotamer library

9.1.7 THR

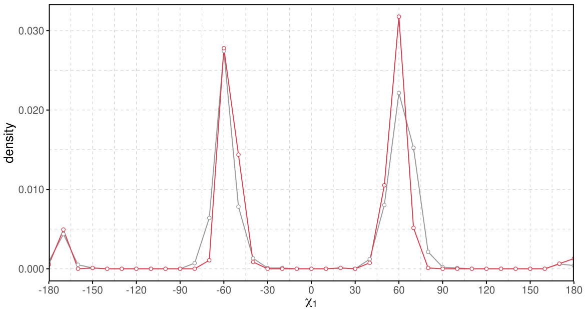

THR – χ_1 dihedral angles

Figure S40: *rotag* rotamer library

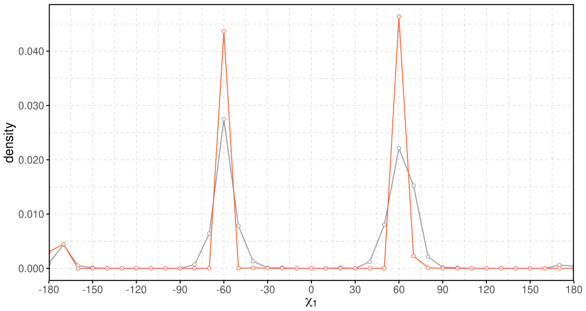

THR – χ_1 dihedral angles

Figure S41: Dunbrack (BBDEP) rotamer library

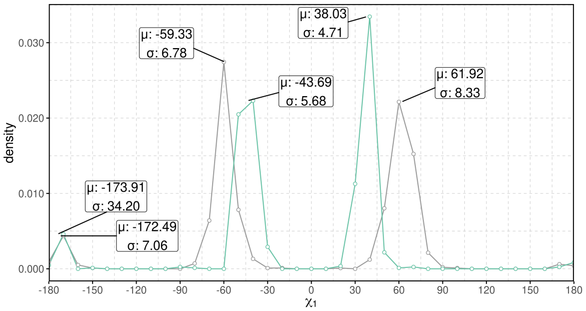

THR – χ_1 dihedral angles

Figure S42: Dynameomics (BBDEP) rotamer library

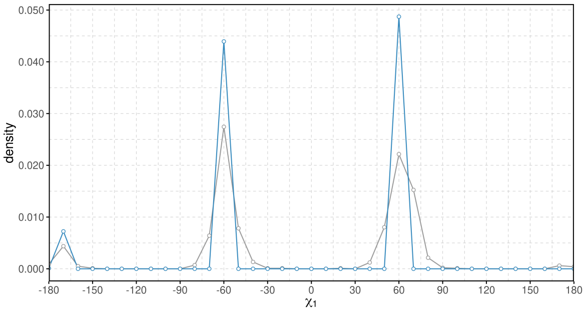

THR – χ_1 dihedral angles

Figure S43: Ultimate (BBIND) rotamer library

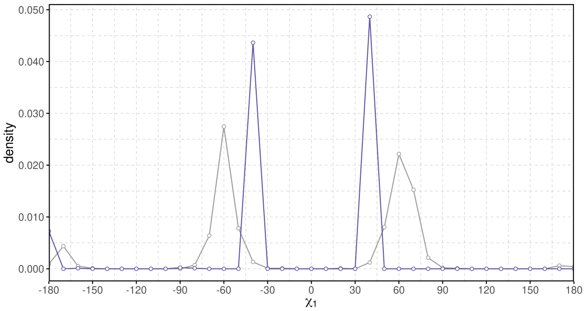

THR – χ_1 dihedral angles

Figure S44: Dynameomics (BBIND) rotamer library

9.1.8 TYR

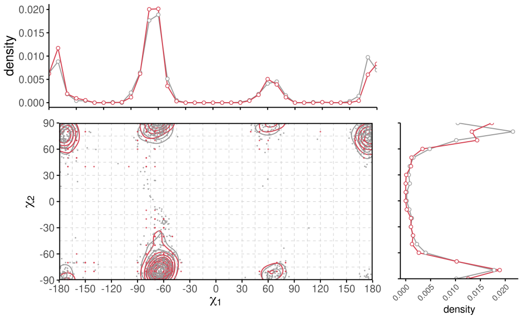

TYR – χ_1 and χ_2 dihedral angles

Figure S45: *rotag* rotamer library

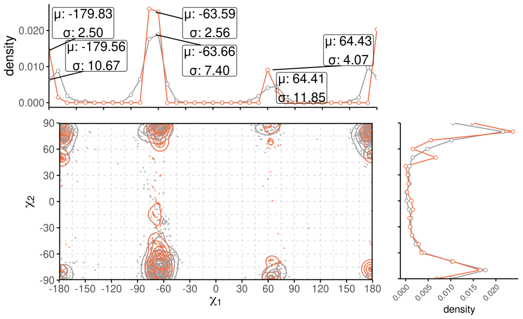

TYR – χ_1 and χ_2 dihedral angles

Figure S46: Dunbrack (BBDEP) rotamer library

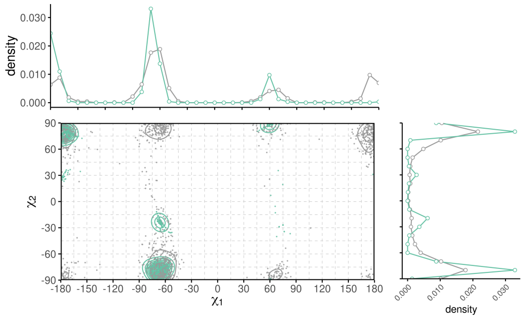

TYR – χ_1 and χ_2 dihedral angles

Figure S47: Dynameomics (BBDEP) rotamer library

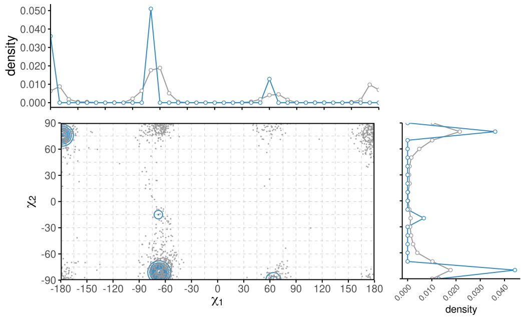

TYR – χ_1 and χ_2 dihedral angles

Figure S48: Ultimate (BBIND) rotamer library

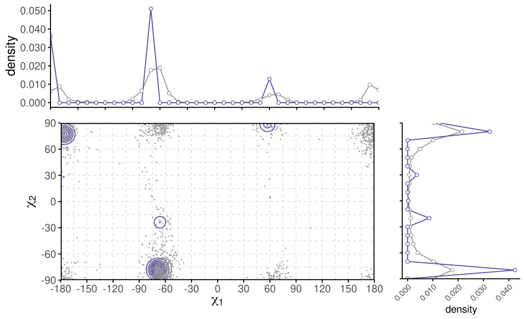

TYR – χ_1 and χ_2 dihedral angles

Figure S49: Dynameomics (BBIND) rotamer library

9.2 Raw data set results

The raw analysis data of the rest of data sets including 13097 can be found in Supplementary File *rotamer-library-bc-dihedral-angles.csv* of *Supplementary-Data.zip* archive.
